# Phenotypes and Clinical Outcome of Heart Failure With Preserved Ejection Fraction Patients in China: Findings From the Chinese Cardiovascular Association Database‐Heart Failure Center Registry

**DOI:** 10.1002/mco2.70642

**Published:** 2026-02-19

**Authors:** Shuai Yuan, Zhonglei Xie, Xiaotong Cui, Shun Yao, Yamei Xu, Yanyan Wang, Yu Song, Kai Hu, Yugang Dong, Yuhua Liao, Weimin Li, Xinli Li, Jiefu Yang, Jingmin Zhou, Junbo Ge

**Affiliations:** ^1^ Department of Cardiology Zhongshan Hospital Fudan University Shanghai Institute of Cardiovascular Diseases Shanghai China; ^2^ Department of Cardiology Shanghai Geriatric Medical Center Zhongshan Hospital Fudan University Shanghai China; ^3^ Institutes of Biomedical Sciences, Fudan University Shanghai China; ^4^ Department of Cardiology The First Affiliated Hospital of Sun Yat‐Sen University Guangzhou China; ^5^ Department of Cardiology Union Hospital Tongji Medical College Huazhong University of Science and Technology Wuhan China; ^6^ Department of Cardiology The First Affiliated Hospital of Harbin Medical University Harbin China; ^7^ Department of Cardiology The First Affiliated Hospital of Nanjing Medical University Nanjing China; ^8^ Department of Cardiology Beijing Hospital Beijing China

**Keywords:** clinical outcomes, etiological phenotypes, heart failure with preserved ejection fraction, risk factors, treatment strategies

## Abstract

Heart failure with preserved ejection fraction (HFpEF) is a highly heterogeneous syndrome that poses challenges for therapeutic development and contributes to suboptimal patient outcomes. The phenotypic classification of patients with HFpEF to guide etiology‐specific therapeutic strategies represents a rational approach to address the current dilemma. However, the clinical outcomes of HFpEF under different etiological classifications remain poorly understood. Here, we assessed the clinical outcomes of HFpEF patients across different etiological phenotypes, based on a novel classification system comprising five categories: vascular‐related, cardiomyopathy‐related, right heart/pulmonary‐related, valvular/rhythm‐related, and extracardiac disease‐related HFpEF. Data from the Chinese Cardiovascular Association Database‐Heart Failure Center Registry (2017–2021) were analyzed, including 51,466 hospitalized HFpEF patients with 1‐year follow‐up. Significant differences in baseline characteristics and clinical outcomes were observed among phenotypes. Patients with right heart/pulmonary‐related, valvular/rhythm‐related, and extracardiac disease‐related HFpEF showed a higher incidence of adverse outcomes. Specifically, the right heart/pulmonary‐related and valvular/rhythm‐related phenotypes were associated with increased heart failure rehospitalization, while extracardiac disease‐related HFpEF was linked to higher cardiovascular mortality. Prognostic risk factors also varied across phenotypes. In conclusion, 1‐year outcomes exhibit significant variations across HFpEF phenotypic subgroups. Future studies should explore whether phenotype‐specific personalized treatment strategies can improve clinical outcomes, especially in high‐risk phenotypes.

## Graphical abstract

Structured graphical abstract‐text element

Key Question

It remains unclear whether patients with heart failure with preserved ejection fraction (HFpEF) in different phenotypes also have various clinical outcomes.

Key Finding

Significant differences existed in baseline characteristics and outcomes among different phenotypes; patients with HFpEF‐3 (right heart and pulmonary‐related HFpEF), HFpEF‐4 (valvular‐ and rhythm‐related HFpEF), and HFpEF‐5 (extracardiac disease‐related HFpEF) exhibited a high incidence of adverse outcomes.

Take‐Home Message

One‐year outcome differs among HFpEF patients with various phenotypes. Future studies are warranted to validate whether personalized treatment strategies based on HFpEF phenotypes could improve the individual outcome of HFpEF patients.

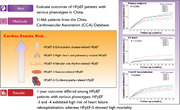

## 1 Introduction

Heart failure (HF) is a clinical syndrome challenged by high morbidity, mortality, and healthcare expenditure both in developed countries and in China [1, 2]. The global prevalence of HF is estimated to be 1%–3% of the general adult population, corresponding to approximately 56.2 million individuals worldwide [3]. Moreover, the prevalence of HF continues to increase, with projections indicating a 46% rise in the number of patients from 2012 to 2030 [4]. In China, the population of HF patients is estimated to be approximately 12.1 million, posing a substantial threat to public health and imposing a considerable economic burden [5].

HF is classified into three types based on the left ventricular ejection fraction (LVEF), which are reduced ejection fraction (HFrEF), mildly reduced ejection fraction (HFmrEF), and preserved ejection fraction (HFpEF) [6]. HFpEF is the main type of HF, accounting for approximately 50% of the overall HF cases [7]. However, the syndrome of HFpEF has not responded well to the standard treatment approach, which achieved better effects in stabilizing and reversing HFrEF over the last two decades, till the positive results achieved by angiotensin‐neprilysin inhibitor saubitril‐valsartan [8, 9] and the sodium–glucose cotransporter 2 inhibitor (SGLT2i) [10, 11, 12]. The heterogeneity in the etiology and pathophysiology of HFpEF, either alone or in combination, may partly contribute to the unsatisfactory therapeutic approaches for HFpEF patients. Indeed, several clinical trials demonstrated that some HFpEF patients benefited from etiology‐oriented therapies [13, 14]. Therefore, individualized therapy options according to the phenotype of HFpEF patients might be a more accurate and ideal strategy to overcome the current treatment challenge.

Several studies based on latent class analysis (LCA) have identified subgroups of HFpEF that exhibit unique clinical characteristics associated with clinical outcomes [15, 16, 17]. Studies are needed to validate whether targeting the identified clinical factors might offer better outcome results. However, most of these studies were based on the re‐analysis of data from large clinical trials, which were unavoidably related to the potential bias in patient selection. Recently, we advocated for a shift away from the “one‐size‐fits‐all” approach in HFpEF toward an etiology‐oriented treatment principle, proposing a novel etiological classification system for this condition, which includes: (i) HFpEF‐1: Vascular‐related HFpEF; (ii) HFpEF‐2: Cardiomyopathy‐related HFpEF; (iii) HFpEF‐3: Right heart and pulmonary‐related HFpEF; (iv) HFpEF‐4: Valvular‐ and rhythm‐related HFpEF; and (v) HFpEF‐5: Extracardiac disease‐related HFpEF [18]. Nevertheless, it remains unclear whether HFpEF patients with various phenotypes based on this phenotyping method also have various clinical outcomes.

The Chinese Cardiovascular Association (CCA) Database‐Heart Failure Center Registry is a nationwide, multicenter study of HF patients in China [19]. Utilizing this platform, we assessed the clinical characteristics and 1‐year outcome of hospitalized HFpEF patients with various phenotypes, and evaluated the risk factors of outcome in the five phenotypes.

Our results demonstrated that baseline characteristics and clinical outcomes differed among the five phenotypes. HFpEF‐3, HFpEF‐4, and HFpEF‐5 showed a higher incidence of adverse outcomes. Specifically, HFpEF‐3 and HFpEF‐4 were associated with increased heart failure rehospitalization, while HFpEF‐5 was linked to higher cardiovascular mortality. Prognostic risk factors also varied across phenotypes. Etiology‐based phenotyping offers an effective strategy to address the current therapeutic challenges in HFpEF.

## 2 Results

**FIGURE 1:**
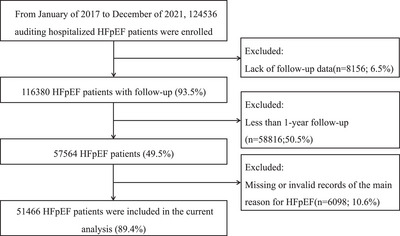
The flowchart of the study: 51,466 HFpEF (heart failure with preserved ejection fraction) patients were included in the final analysis.

A total of 124,536 HFpEF patients were registered from 481 certified secondary or tertiary hospitals in mainland China between January 2017 and December 2021. After excluding those who were lost to follow‐up (n = 8156), less than 1‐year follow‐up after discharge (*n* = 58,816), or missing or invalid records of the main reason for HF (*n* = 6098), 51,466 HFpEF patients were included in the final analysis (Figure 1). There are no significant differences between the included and excluded cohorts in baseline characteristics (SMD < 0.10) (Tables S1 and S2).

### 2.1 Baseline Clinical Characteristics of Different Phenotypes

The median age of included patients was 74.0 years, and 49.8% were women. The median BMI was 23.7 kg/m^2^, with a prevalence of obesity at 10.0%. The median systolic blood pressure (SBP) was 129.0 mmHg, and the median heart rate was 79.0 bpm. NYHA III–IV patients accounted for 43.7%; 17.7% of patients had HHF in the last 1 year before index admission. Median LVEF was 59.0%. The leading three inducements of HHF after discharge were CHD (29.5%), infection (14.1%), and arrhythmia (9.7%). The most prevalent comorbidity was hypertension (68.4%), followed by CHD (40.8%), AF (39.9%), DM (29.6%), anemia (27.2%), and CKD (14.0%).

**HFpEF‐1** was the largest population (78.2%), followed by HFpEF‐4 (16.3%), HFpEF‐2 (3.8%), HFpEF‐5 (1.3%), and HFpEF‐3 (0.4%). Compared to other phenotypes, HFpEF‐1 patients were much older, had higher BMI and blood pressure, but a slower heart rate and less HHF in the previous 12 months. Both the eGFR and NT‐proBNP levels in the HFpEF‐1 group were relatively lower, but the proportions of patients with various comorbidities were significantly higher. The revascularization rate (percutaneous coronary intervention [PCI] 20.8%, coronary artery bypass grafting [CABG] 1.2%) and use of angiotensin converting enzyme inhibitor (ACEI) or angiotensin receptor antagonist (ARB) (58.8%), calcium channel blocker (CCB) (26.7%), antiplatelets (69.7%), statin (81.7%), nitrate (34.4%), and anti‐DM drugs (28.0%) were highest in HFpEF‐1 group, while the use rate of diuretics was lowest compared to other groups.

In the **HFpEF‐2** group, prior hospitalization within the last 1 year was more common (23.0%), and patients also exhibited more significant structural changes in the heart, characterized by an increase in left ventricular end‐diastolic diameter (LVEDD) (median 51 mm). Additionally, the proportion of device therapy, including cardiac resynchronization therapy (CRT) (3.9%), implantable cardioverter defibrillator (ICD) (2.2%), and pacemaker (7.0%), was higher compared to the other phenotypes. The usage of medication in this group showed a higher proportion of angiotensin receptor neprilysin inhibitor (ARNI) (24.7%) and beta‐blocker drugs (78.4%).

**HFpEF‐3** patients were younger (median age 59.0 years), and women were the majority of them, which accounted for 63.6%. They had the fewest smokers (17.7%), the lowest BMI (median 21.8 kg/m^2^) and SBP/DBP (median 112.0/68.0 mmHg), and had fewer comorbidities. But their baseline RHR (median 85 bpm) and NT‐proBNP (median 1953.9pg/mL) were high.

The proportion of AF patients was highest in **HFpEF‐4** (68.2%). In this group, more patients were classified as NYHA (New York Heart Association) Class III–IV (51.4%), and they received a higher rate of treatment with aldosterone antagonists (76.1%), diuretics (83.0%), oral anticoagulants (55.0%), and digitalis (25.9%) compared to other groups.

**HFpEF‐5** comprised more NYHA I–II patients (70.8%); patients in this group exhibited higher rates of smoking (30.8%) and had comorbidities such as COPD (37.3%), anemia (29.1%), anxiety (5.3%), depression (5.3%), and thyroid disease (14.7%) (Table 1).

**TABLE 1:** Baseline clinical characteristics of five phenotypes.

		Overall	HFpEF‐1	HFpEF‐2	HFpEF‐3	HFpEF‐4	HFpEF‐5	*p*‐value
***n* (%)**		51,466	40,224 (78.2%)	1979 (3.8%)	198 (0.39%)	8387 (16.3%)	678 (1.3%)	
Age (median [IQR])		74.00 [66.00, 82.00]	75.00 [67.00, 82.00]	67.00 [55.00, 76.00]	59.00 [46.00, 70.00]	71.00 [62.00, 79.00]	73.00 [65.00, 81.00]	<0.001
Sex (%)	Female	25,626 (49.8)	19,310 (48.0)	799 (40.4)	126 (63.6)	5041 (60.1)	350 (51.6)	<0.001
	Male	25,818 (50.2)	20,895 (51.9)	1178 (59.5)	72 (36.4)	3345 (39.9)	328 (48.4)	
	Unknown	22 (0.0)	19 (0.0)	2 (0.1)	0 (0.0)	1 (0.0)	0 (0.0)	
Occupation (%)	Agricultural, manufacturing, services, or sales workers	12,500 (24.3)	9290 (23.1)	664 (33.6)	82 (41.4)	2212 (26.4)	252 (37.2)	<0.001
	Housework, retired, unemployed, or other occupations	34,543 (67.1)	27,277 (67.8)	1173 (59.3)	115 (58.1)	5563 (66.3)	415 (61.2)	
	Managers or professionals	725 (1.4)	564 (1.4)	56 (2.8)	1 (0.5)	95 (1.1)	9 (1.3)	
	Unknown	3698 (7.2)	3093 (7.7)	86 (4.3)	0 (0.0)	517 (6.2)	2 (0.3)	
Alcohol consumption (%)	Previous or current	8782 (17.1)	7024 (17.5)	470 (23.7)	28 (14.1)	1128 (13.4)	132 (19.5)	<0.001
Previous or current smoker (%)	Previous or current	13,712 (26.6)	11,204 (27.9)	598 (30.2)	35 (17.7)	1666 (19.9)	209 (30.8)	<0.001
BMI (median [IQR])		23.74 [21.26, 26.35]	24.03 [21.51, 26.57]	23.73 [21.21, 26.64]	21.75 [19.15, 24.61]	22.66 [20.20, 25.31]	22.38 [19.55, 25.39]	<0.001
SBP (median [IQR])		129.00 [116.00, 143.00]	130.00 [119.00, 145.00]	120.00 [110.00, 135.00]	112.00 [102.00, 124.75]	121.00 [110.00, 135.00]	121.00 [110.00, 133.00]	<0.001
DBP (median [IQR])		74.00 [66.00, 82.00]	75.00 [67.00, 83.00]	72.00 [65.00, 80.00]	68.00 [60.00, 74.00]	71.00 [64.00, 80.00]	70.00 [64.00, 78.00]	<0.001
RHR (median [IQR])		79.00 [68.00, 92.00]	78.00 [68.00, 90.00]	78.00 [68.00, 92.00]	85.00 [75.00, 101.75]	82.00 [70.00, 98.00]	84.00 [72.00, 98.00]	<0.001
HHF in previous 12 months (%)		9133 (17.7)	6851 (17.0)	456 (23.0)	38 (19.2)	1667 (19.9)	121 (17.8)	<0.001
HF main inducement (%)	Arrhythmia	4985 (9.7)	2919 (7.3)	288 (14.6)	28 (14.1)	1640 (19.6)	110 (16.2)	<0.001
	Coronary artery disease	15,190 (29.5)	14,731 (36.6)	130 (6.6)	4 (2.0)	298 (3.6)	27 (4.0)	
	Infection	7277 (14.1)	5059 (12.6)	319 (16.1)	42 (21.2)	1630 (19.4)	227 (33.5)	
	Other	10,059 (19.5)	6043 (15.0)	795 (40.2)	103 (52.0)	2856 (34.1)	262 (38.6)	
	Poor adherence to medication	1960 (3.8)	1335 (3.3)	155 (7.8)	11 (5.6)	438 (5.2)	21 (3.1)	
	Uncontrolled hypertension	3305 (6.4)	3016 (7.5)	67 (3.4)	3 (1.5)	200 (2.4)	19 (2.8)	
	Volume overload	652 (1.3)	347 (0.9)	51 (2.6)	7 (3.5)	235 (2.8)	12 (1.8)	
	Unknown	8038 (15.6)	6774 (16.8)	174 (8.8)	0 (0.0)	1090 (13.0)	0 (0.0)	
NYHA (%)	III–IV	22,472 (43.7)	17,092 (42.5)	791 (40.0)	83 (41.9)	4309 (51.4)	197 (29.1)	<0.001
Glucose (median [IQR])		5.70 [4.93, 7.11]	5.80 [4.99, 7.33]	5.35 [4.79, 6.34]	5.29 [4.50, 6.33]	5.40 [4.80, 6.43]	5.53 [4.80, 6.86]	<0.001
Glycosylated hemoglobin (median [IQR])		6.18 [5.69, 7.10]	6.30 [5.70, 7.40]	6.00 [5.50, 6.60]	6.00 [5.60, 6.70]	5.90 [5.50, 6.40]	5.90 [5.50, 6.60]	<0.001
Hemoglobin (median [IQR])		128.00 [111.00, 148.00]	128.00 [111.00, 147.00]	138.00 [119.00, 176.00]	142.00 [121.00, 880.00]	125.00 [108.00, 146.00]	132.00 [108.00, 815.00]	<0.001
UA (median [IQR])		386.00 [305.00, 486.48]	381.00 [302.00, 477.40]	407.00 [318.00, 514.00]	432.00 [338.00, 571.40]	400.00 [313.00, 509.18]	401.00 [306.95, 526.00]	<0.001
Creatinine (median [IQR])		84.15 [67.40, 112.00]	85.40 [68.00, 114.10]	81.00 [66.80, 106.50]	70.30 [58.55, 89.00]	80.70 [65.00, 104.00]	81.95 [64.00, 124.00]	<0.001
eGFR (median [IQR])		77.89 [54.16, 101.02]	76.73 [52.87, 99.88]	85.35 [59.50, 107.28]	96.83 [74.20, 122.66]	80.91 [59.50, 103.23]	78.56 [48.78, 111.46]	<0.001
NTproBNP (median [IQR])		1593.00 [607.48, 3865.00]	1544.00 [563.00, 3854.25]	1457.00 [512.75, 4001.50]	1953.85 [682.45, 4518.00]	1765.00 [807.20, 3830.00]	1884.00 [701.00, 4336.50]	<0.001
Sodium (median [IQR])		140.00 [137.50, 142.00]	140.00 [137.70, 142.00]	140.00 [138.00, 142.00]	139.10 [136.90, 142.00]	139.50 [137.00, 142.00]	139.90 [137.10, 142.00]	<0.001
Potassium (median [IQR])		4.07 [3.77, 4.40]	4.07 [3.76, 4.40]	4.08 [3.79, 4.40]	4.10 [3.79, 4.41]	4.09 [3.80, 4.40]	4.11 [3.78, 4.47]	0.017
QOLQ (median [IQR])		70.00 [55.00, 80.00]	70.00 [60.00, 80.00]	70.00 [60.00, 80.00]	67.50 [48.00, 75.75]	65.00 [50.00, 76.00]	71.00 [50.00, 75.00]	<0.001
MLHF (median [IQR])		40.00 [20.00, 68.00]	40.00 [20.00, 70.00]	37.00 [20.75, 62.00]	38.00 [24.00, 56.00]	40.00 [21.00, 65.00]	43.00 [25.00, 60.00]	0.591
LVEF (median [IQR])		59.00 [55.00, 64.00]	59.00 [55.00, 64.00]	58.00 [53.00, 62.00]	60.50 [56.00, 65.72]	58.00 [54.00, 63.00]	59.00 [55.00, 65.00]	<0.001
LVEDD (median [IQR])		48.00 [43.00, 52.00]	47.00 [43.30, 52.00]	51.00 [46.00, 58.00]	43.00 [37.55, 48.50]	48.00 [43.00, 53.00]	45.00 [40.00, 49.40]	<0.001
6‐minute walk distance (median [IQR])		350.00 [261.50, 430.00]	350.00 [260.00, 425.00]	380.00 [300.00, 470.00]	350.00 [320.00, 425.00]	357.50 [259.50, 446.00]	344.00 [256.00, 425.00]	0.035
Obesity (%)		5150 (10.0)	4247 (10.6)	219 (11.1)	14 (7.1)	612 (7.3)	58 (8.6)	<0.001
Hypertension (%)		35,226 (68.4)	30,404 (75.6)	873 (44.1)	52 (26.3)	3566 (42.5)	331 (48.8)	<0.001
DM (%)		15,218 (29.6)	13,475 (33.5)	325 (16.4)	19 (9.6)	1270 (15.1)	129 (19.0)	<0.001
MI (%)		11,219 (21.8)	10,998 (27.3)	52 (2.6)	2 (1.0)	148 (1.8)	19 (2.8)	<0.001
CHD (%)		20,980 (40.8)	19,669 (48.9)	288 (14.6)	12 (6.1)	880 (10.5)	131 (19.3)	<0.001
AF (%)		20,548 (39.9)	13,648 (33.9)	860 (43.5)	75 (37.9)	5720 (68.2)	245 (36.1)	<0.001
Stroke/TIA (%)		8675 (16.9)	7310 (18.2)	171 (8.6)	11 (5.6)	1111 (13.2)	72 (10.6)	<0.001
PAD (%)		5908 (11.5)	4744 (11.8)	179 (9.0)	17 (8.6)	866 (10.3)	102 (15.0)	<0.001
Dyslipidemia (%)		8364 (16.3)	7355 (18.3)	218 (11.0)	8 (4.0)	743 (8.9)	40 (5.9)	<0.001
COPD (%)		5197 (10.1)	4160 (10.3)	145 (7.3)	14 (7.1)	625 (7.5)	253 (37.3)	<0.001
OSAHS (%)		199 (0.4)	157 (0.4)	18 (0.9)	1 (0.5)	21 (0.3)	2 (0.3)	<0.001
CKD (%)		7192 (14.0)	6025 (15.0)	195 (9.9)	11 (5.6)	865 (10.3)	96 (14.2)	<0.001
Anemia (%)		14,004 (27.2)	10,876 (27.0)	371 (18.7)	29 (14.6)	2531 (30.2)	197 (29.1)	<0.001
Anxiety (%)		610 (1.2)	397 (1.0)	23 (1.2)	7 (3.5)	147 (1.8)	36 (5.3)	<0.001
Depression (%)		559 (1.1)	365 (0.9)	22 (1.1)	7 (3.5)	129 (1.5)	36 (5.3)	<0.001
Malignant tumor (%)		1525 (3.0)	1272 (3.2)	47 (2.4)	2 (1.0)	174 (2.1)	30 (4.4)	<0.001
Thyroid disease (%)		3005 (5.8)	2189 (5.4)	125 (6.3)	11 (5.6)	580 (6.9)	100 (14.7)	<0.001
CRT (%)		185 (0.4)	97 (0.2)	77 (3.9)	0 (0.0)	11 (0.1)	0 (0.0)	<0.001
ICD (%)		149 (0.3)	89 (0.2)	44 (2.2)	0 (0.0)	16 (0.2)	0 (0.0)	<0.001
Pacemaker (%)		2278 (4.4)	1661 (4.1)	138 (7.0)	4 (2.0)	452 (5.4)	23 (3.4)	<0.001
PCI (%)		8577 (16.7)	8375 (20.8)	55 (2.8)	2 (1.0)	130 (1.6)	15 (2.2)	<0.001
CABG (%)		551 (1.1)	475 (1.2)	4 (0.2)	0 (0.0)	71 (0.8)	1 (0.1)	<0.001
ACEI (%)		13,349 (25.9)	10,824 (26.9)	578 (29.2)	24 (12.1)	1822 (21.7)	101 (14.9)	<0.001
ARB (%)		15,137 (29.4)	12,851 (31.9)	459 (23.2)	18 (9.1)	1683 (20.1)	126 (18.6)	<0.001
ARNI (%)		6880 (13.4)	5125 (12.7)	488 (24.7)	25 (12.6)	1132 (13.5)	110 (16.2)	<0.001
Beta blocker (%)		37,018 (71.9)	29,404 (73.1)	1551 (78.4)	104 (52.5)	5566 (66.4)	393 (58.0)	<0.001
MRA (%)		32,115 (62.4)	23,715 (59.0)	1440 (72.8)	150 (75.8)	6384 (76.1)	426 (62.8)	<0.001
SGLT2i (%)		1309 (2.5)	1117 (2.8)	40 (2.0)	3 (1.5)	128 (1.5)	21 (3.1)	<0.001
Diuretics (%)		35,280 (68.6)	26,183 (65.1)	1462 (73.9)	164 (82.8)	6961 (83.0)	510 (75.2)	<0.001
Digoxin (%)		6643 (12.9)	3994 (9.9)	366 (18.5)	41 (20.7)	2173 (25.9)	69 (10.2)	<0.001
Ivabradine (%)		758 (1.5)	629 (1.6)	49 (2.5)	4 (2.0)	60 (0.7)	16 (2.4)	<0.001
Antiarrhythmics (%)		4004 (7.8)	3041 (7.6)	194 (9.8)	13 (6.6)	723 (8.6)	33 (4.9)	<0.001
Antiplatelets (%)		30,875 (60.0)	28,034 (69.7)	677 (34.2)	33 (16.7)	1939 (23.1)	192 (28.3)	<0.001
Statins (%)		37,728 (73.3)	32,858 (81.7)	987 (49.9)	43 (21.7)	3536 (42.2)	304 (44.8)	<0.001
Oral anticoagulants (%)		15,173 (29.5)	9622 (23.9)	670 (33.9)	63 (31.8)	4613 (55.0)	205 (30.2)	<0.001
CCB (%)		12,074 (23.5)	10,737 (26.7)	212 (10.7)	10 (5.1)	1010 (12.0)	105 (15.5)	<0.001
Nitrate (%)		15,526 (30.2)	13,793 (34.3)	300 (15.2)	26 (13.1)	1303 (15.5)	104 (15.3)	<0.001
Oral antidiabetics (%)		12,713 (24.7)	11,266 (28.0)	268 (13.5)	18 (9.1)	1063 (12.7)	98 (14.5)	<0.001
Inotropes (%)		6410 (12.5)	4566 (11.4)	294 (14.9)	28 (14.1)	1448 (17.3)	74 (10.9)	<0.001

Abbreviations: ACEI, angiotensin‐converting enzyme inhibitor; AF, atrial fibrillation; ARB, angiotensin receptor blocker; ARNI, angiotensin receptor–neprilysin inhibitor; BMI, body mass index; CABG, coronary artery bypass grafting; CHD, coronary heart disease; CKD, chronic kidney disease; COPD, chronic obstructive pulmonary disease; CRT, cardiac resynchronization therapy; DBP, diastolic blood pressure; DM, diabetes mellitus; eGFR, estimated glomerular filtration rate; HF, heart failure; HHF, heart failure hospitalization; ICD, implantable cardioverter defibrillator; LVEDD, left ventricular end‐diastolic dimension; LVEF, left ventricular ejection fraction; MI, myocardial infarction; MLHF, Minnesota Living With Heart Failure; MRA, mineralocorticoid receptor antagonist; NT‐proBNP, N‐terminal pro‐B‐type natriuretic peptide; NYHA, New York Heart Association; OSAHS, obstructive sleep apnea‐hypopnea syndrome; PAD, peripheral arterial disease; PCI, percutaneous coronary intervention; QOLQ, Quality of Life Questionnaire; RHR, rest heart rate; SBP, systolic blood pressure; SGLT2i, sodium–glucose cotransporter 2 inhibitor; TIA, transient ischemic attacks; UA, uric acid.

### 2.2 Relationship Between HFpEF Phenotypes and the Primary Outcome

The rate of primary outcome at 1‐year follow‐up in the overall study participants was 10.6% (11.7 per 100 person‐years), with CV death of 2.5% (2.6 per 100 person‐years) and first time of HHF of 8.3% (9.2 per 100 person‐years), respectively. For the secondary outcome, the rate of all‐cause death at 1‐year follow‐up was 6.5% (6.8 per 100 person‐years), and the rate of first‐time all‐cause hospitalization was 10.3% (11.5 per 100 person‐years) (Table 2).

**TABLE 2:** Outcomes of HFpEF phenotypes at 1‐year follow‐up.

		Total	HFpEF‐1	HFpEF‐2	HFpEF‐3	HFpEF‐4	HFpEF‐5
Primary endpoint	Number of events (%)	5435 (10.6)	4138 (10.3)	173 (8.7)	27 (13.6)	1016 (12.1)	81 (11.9)
	Incidence rate/100 person‐year (95% CI)	11.7 (11.4–12)	11.4 (11.1–11.7)	9.5 (8.2–10.9)	15.4 (10.4–21.6)	13.6 (12.9–14.4)	13.9 (1.2–17)
CV death	Number of events (%)	1269 (2.5)	944 (2.3)	40 (2)	7 (3.5)	235 (2.8)	43 (6.3)
	Incidence rate/100 person‐year (95% CI)	2.6 (2.4–2.7)	2.5 (2.3–2.6)	2.1 (1.5–2.8)	3.7 (1.5–7.5)	2.9 (2.6–3.3)	7.1 (5.2–9.4)
First HHF	Number of events (%)	4284 (8.3)	3281 (8.2)	137 (6.9)	20 (10.1)	807 (9.6)	39 (5.8)
	Incidence rate/100 person‐year (95% CI)	9.2 (9–9.5)	9 (8.7–9.3)	7.5 (6.4–8.8)	11.4 (7.1–17.1)	10.8 (10.1–11.6)	6.7 (4.8–9)
All‐cause death	Number of events (%)	3349 (6.5)	2588 (6.4)	97 (4.9)	14 (7.1)	555 (6.6)	95 (14)
	Incidence rate/100 person‐year (95% CI)	6.8 (6.6–7)	6.7 (6.5–7)	5.1 (4.1–6.2)	7.4 (4.1–12.2)	6.9 (6.4–7.5)	15.7 (12.9–18.8)
Non‐CV death	Number of events (%)	1312 (2.5)	998 (2.5)	44 (2.2)	6 (3)	221 (2.6)	43 (6.3)
	Incidence rate/100 person‐year (95% CI)	2.7 (2.5–2.8)	2.6 (2.4–2.8)	2.3 (1.7–3.1)	3.2 (1.2–6.8)	2.8 (2.4–3.1)	7.1 (5.2–9.4)
First, all‐cause hospitalization	Number of events (%)	5319 (10.3)	4148 (10.3)	170 (8.6)	22 (11.1)	929 (11.1)	50 (7.4)
	Incidence rate/100 person‐year (95% CI)	11.5 (11.3–11.8)	11.5 (11.2–11.8)	9.4 (8.1–10.8)	12.6 (8–18.4)	12.5 (11.8–13.3)	8.6 (6.5–11.2)
CV hospitalization	Number of events (%)	4627 (9)	3582 (8.9)	144 (7.3)	20 (10.1)	841 (10)	40 (5.9)
	Incidence rate/100 person‐year (95% CI)	10 (9.8–10.3)	9.9 (9.6–10.2)	7.9 (6.7–9.3)	11.4 (7.1–17.1)	11.3 (10.6–12.1)	6.9 (5–9.3)

Abbreviations: CV death, cardiovascular‐related death; CV hospitalization, cardiovascular‐related hospitalization; First HHF, first time of heart failure hospitalization; No‐CV death, not cardiovascular‐related death.

**FIGURE 2:**
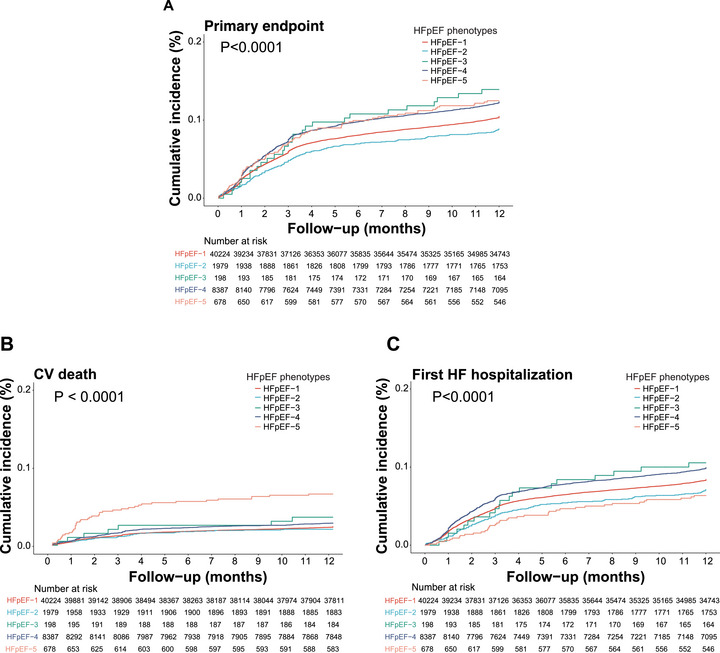
Cumulative incidence of primary outcome at 1‐year follow‐up in the five phenotypes. (A) The cumulative incidence of primary endpoint in the five phenotypes. (B) The cumulative incidence of cardiovascular (CV) death in the five phenotypes. (C) The cumulative incidence of hospitalization for heart failure (HHF) in the five phenotypes.

As shown in Tables 2 and 3 and Figure 2, the primary outcomes of five phenotypes showed significant differences (*p* < 0.0001). Patients in HFpEF‐2 have the best prognosis, followed by HFpEF‐1, while patients in HFpEF‐3, HFpEF‐4, and HFpEF‐5 have relatively poor prognosis (*p* < 0.0001; Figure 2A–C). Compared to HFpEF‐2, HFpEF‐3 (HR 1.70, 95% CI: 1.05–2.75), HFpEF‐4 (HR 1.31, 95% CI: 1.05–1.63), and HFpEF‐5 patients (HR 1.46, 95% CI: 1.06–2.00) showed higher risk (Table 3). Extreme Scenario Analysis in both worst‐case (all lost‐to‐follow‐up assigned as events) and best‐case (all assumed event‐free) scenarios, effect estimates remained directionally consistent with the primary findings. No qualitative changes in the associations were observed, indicating that the results are robust to potential attrition‐related bias (Tables S3 and S4).

**TABLE 3:** Clinical outcome at 1‐year follow‐up in 5 phenotypes.

Phenotype	HR (95%CI)	P‐value
Primary endpoint		
HFpEF‐2	Reference	
HFpEF‐1	1.12 (0.91‐1.38)	0.276
HFpEF‐3	1.70 (1.05‐2.75)	0.032
HFpEF‐4	1.31 (1.05‐1.63)	0.017
HFpEF‐5	1.46 (1.06‐2.00)	0.02
CV death		
HFpEF‐2	Reference	
HFpEF‐1	1.25 (0.82‐1.90)	0.305
HFpEF‐3	1.37 (0.47‐3.96)	0.566
HFpEF‐4	1.38 (0.89‐2.13)	0.155
HFpEF‐5	2.75 (1.59‐4.75)	〈0.001
First HHF		
HFpEF‐2	Reference	
HFpEF‐1	1.10 (0.87‐1.40)	0.437
HFpEF‐3	1.78 (1.03‐3.06)	0.038
HFpEF‐4	1.31 (1.02‐1.69)	0.034
HFpEF‐5	1.06 (0.71‐1.58)	0.776
All‐cause death		
HFpEF‐2	Reference	
HFpEF‐1	1.14 (0.88‐1.49)	0.323
HFpEF‐3	1.48 (0.75‐2.89)	0.256
HFpEF‐4	1.18 (0.90‐1.57)	0.235
HFpEF‐5	1.96 (1.38‐2.80)	〈0.001
First all‐cause hospitalization		
HFpEF‐2	Reference	
HFpEF‐1	1.10 (0.89‐1.36)	0.357
HFpEF‐3	1.54 (0.93‐2.55)	0.097
HFpEF‐4	1.22 (0.97‐1.52)	0.083
HFpEF‐5	1.04 (0.73‐1.48)	0.841
Non‐CV death		
HFpEF‐2	Reference	
HFpEF‐1	0.92 (0.63‐1.35)	0.674
HFpEF‐3	1.58 (0.61‐4.09)	0.349
HFpEF‐4	1.03 (0.68‐1.54)	0.903
HFpEF‐5	1.90 (1.14‐3.17)	0.014
First CV hospitalization		
HFpEF‐2	Reference	
HFpEF‐1	1.15 (0.91‐1.45)	0.231
HFpEF‐3	1.72 (1.01‐2.91)	0.044
HFpEF‐4	1.30 (1.02‐1.65)	0.034
HFpEF‐5	1.01 (0.68‐1.49)	0.956

CV death, cardiovascular‐related death; First HHF, first time of heart failure hospitalization; No‐CV death, not cardiovascular‐related death; CV hospitalization, cardiovascular related hospitalization

The proportion of missing values for covariates included in the multivariable models was low (<10% for all variables) (Table S5). Multiple imputation analyses yielded results that were consistent with the complete‐case analyses (Table S6).

### 2.3 Relationship Between HFpEF Phenotypes and Secondary Outcomes

With regard to CV death, HFpEF patients in HFpEF‐5 displayed the highest magnitude of risk compared to HFpEF‐2 (HR 2.75, 95% CI: 1.59–4.75), followed by patients in HFpEF‐4 (HR 1.38, 95% CI: 0.89–2.13), while the other phenotypes demonstrated a similar probability of CV death. Compared to HFpEF‐2, patients in HFpEF‐5 (HR 1.06, 95% CI: 0.71–1.58) showed a lower risk of first time of HHF, while patients in HFpEF‐4 showed significantly higher risk for HHF (HR 1.31, 95% CI: 1.02–1.69) (Figure 2B,C).

**FIGURE 3:**
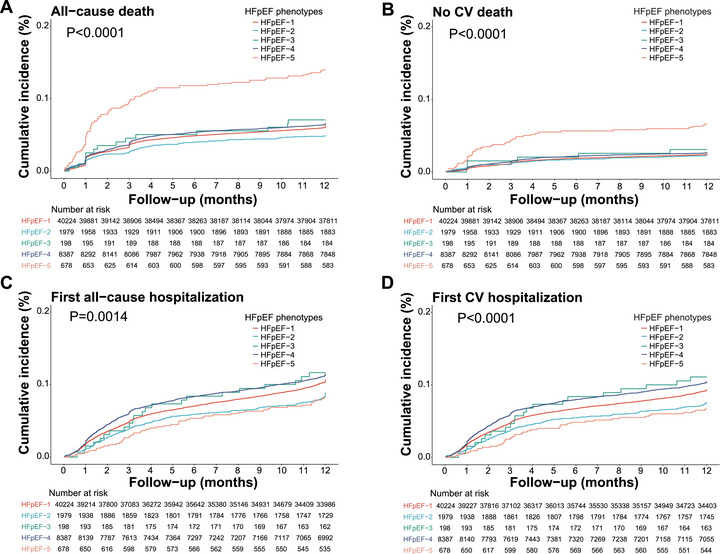
Cumulative incidence of secondary outcome at 1‐year follow‐up in the five phenotypes. (A) The cumulative incidence of all‐cause death in the five phenotypes. (B) The cumulative incidence of death not related to cardiovascular disease (No CV death). (C) The cumulative incidence first time of all‐cause hospitalization (first all‐cause hospitalization) in the five phenotypes. (D) The cumulative incidence first time of cardiovascular‐related hospitalization (first CV hospitalization) in the five phenotypes.

Compared with HFpEF‐2, patients in HFpEF‐5 exhibited a higher risk of all‐cause death (HR 1.96, 95% CI: 1.38–2.80). It was shown that, as to death not related to cardiovascular diseases, patients in HFpEF‐5 still exhibited the highest risk (HR 1.90, 95% CI: 1.14–3.17), while the remaining four phenotypes demonstrated a similar level of risk. In terms of the first occurrence of all‐cause hospitalization, HFpEF‐2 and HFpEF‐5 displayed a lower risk, while other groups did not exhibit a significant difference. Furthermore, when comparing the risk of first cardiovascular‐related hospitalization, we found that it aligned with the risk observed for all‐cause hospitalization (Figure 3A–D). Multiple imputation analyses yielded results that were consistent with the complete‐case analyses (Table S6).

### 2.4 Factors Associated With the Primary Outcome in Five Phenotypes

The risk factors for developing the primary endpoint were analyzed in the overall cohort as well as in the five phenotypic groups (Table 4, Table S7). In multivariate analysis, older age, HHF in the previous 1 year, NYHA III–IV, NTproBNP ≥1600 pg/mL, RHR ≥60 bpm, DM, AF, COPD, CKD, anemia, PCI treatment history, SBP less than 120 mmHg, and serum sodium less than 135 mmol/L were independently associated with the occurrence of the primary endpoint in the whole HFpEF cohort.

**TABLE 4:** Factors associated with primary endpoint by different HFpEF phenotypes in multivariate Cox model at 1‐year follow‐up.

	Total		HFpEF‐1		HFpEF‐2		HFpEF‐3		HFpEF‐4		HFpEF‐5	
	HR (95% CI)	*p*‐value	HR (95% CI)	*p*‐value	HR (95% CI)	*p*‐value	HR (95% CI)	*p*‐value	HR (95% CI)	*p*‐value	HR (95% CI)	*p*‐value
Age	1.02 (1.01–1.02)	<0.001	1.02 (1.01–1.02)	<0.001	1 (0.98–1.01)	0.706			1.02 (1.01–1.02)	<0.001	1.01 (0.99–1.04)	0.265
Female	0.99 (0.92–1.07)	0.806	0.99 (0.91–1.09)	0.903								
HHF in previous 12 months	1.75 (1.61–1.9)	<0.001	1.57 (1.43–1.73)	<0.001	2 (1.3–3.08)	0.002			2.41 (2.01–2.87)	<0.001	2.15 (1.24–3.71)	0.006
NYHA III–IV	1.2 (1.11–1.3)	<0.001	1.27 (1.16–1.39)	<0.001	1.04 (0.69–1.59)	0.838			1.01 (0.84–1.2)	0.952	1.31 (0.77–2.22)	0.319
LVEF ≥60%	0.93 (0.86–1)	0.065	0.93 (0.85–1.02)	0.132	0.73 (0.47–1.14)	0.172						
NT‐proBNP ≥1600 pg/mL	1.69 (1.55–1.84)	<0.001	1.72 (1.56–1.9)	<0.001	1.43 (0.92–2.23)	0.115	2.68 (0.97–7.37)	0.057	1.47 (1.22–1.76)	<0.001	3.15 (1.72–5.79)	<0.001
RHR ≥60 bpm	1.18 (1.01–1.36)	0.032			2.25 (0.82–6.18)	0.115			1.18 (0.84–1.65)	0.343		
Hypertension			0.91 (0.82–1.01)	0.083								
DM	1.12 (1.03–1.22)	0.007	1.15 (1.04–1.26)	0.005			3.19 (1.06–9.56)	0.039	1.1 (0.88–1.39)	0.4		
Obesity	0.98 (0.86–1.12)	0.786	1.04 (0.9–1.2)	0.614								
AF	1.12 (1.03–1.21)	0.007	1.12 (1.02–1.23)	0.018	1.2 (0.78–1.85)	0.395			0.96 (0.8–1.16)	0.68		
MI	1.05 (0.94–1.17)	0.37	1.09 (0.97–1.22)	0.135					1.31 (0.77–2.23)	0.327		
PCI	0.86 (0.76–0.98)	0.022	0.9 (0.79–1.02)	0.1								
COPD	1.24 (1.11–1.39)	<0.001	1.24 (1.1–1.41)	0.001					1.15 (0.86–1.53)	0.346	1.09 (0.65–1.83)	0.748
CKD	1.35 (1.23–1.49)	<0.001	1.41 (1.27–1.57)	<0.001	1.89 (1.08–3.31)	0.025			1.14 (0.88–1.48)	0.307		
Anemia	1.2 (1.11–1.31)	<0.001	1.19 (1.08–1.31)	<0.001	1.37 (0.85–2.22)	0.2			1.14 (0.95–1.38)	0.156	1.67 (0.99–2.81)	0.052
SBP <120 mmHg	1.26 (1.14–1.39)	<0.001	1.21 (1.07–1.36)	0.002			0.32 (0.13–0.79)	0.014	1.38 (1.11–1.72)	0.004	1.16 (0.64–2.1)	0.624
SBP >130 mmHg	1.02 (0.92–1.12)	0.731	1.06 (0.95–1.18)	0.323			0.3 (0.07–1.38)	0.121	0.94 (0.73–1.21)	0.645	0.59 (0.28–1.24)	0.167
Sodium <135 mmol/L	1.18 (1.05–1.32)	0.007	1.23 (1.07–1.4)	0.004	1.11 (0.57–2.16)	0.767			1 (0.78–1.29)	0.992	1.41 (0.68–2.9)	0.357
Sodium >145 mmol/L	1 (0.85–1.19)	0.963	1.14 (0.94–1.37)	0.178	0.86 (0.31–2.38)	0.774			0.51 (0.29–0.91)	0.023	0.72 (0.25–2.03)	0.53
Potassium <3.5 mmol/L	0.99 (0.87–1.13)	0.933	0.97 (0.84–1.13)	0.721								
Potassium >5.5 mmol/L	1.11 (0.82–1.51)	0.491	1.1 (0.78–1.56)	0.59								

Abbreviations: AF, atrial fibrillation; CKD, chronic kidney disease; COPD, chronic obstructive pulmonary disease; DM, diabetes mellitus; HHF, heart failure hospitalization; LVEF, left ventricular ejection fraction; MI, myocardial infarction; NT‐proBNP, N‐terminal pro‐B‐type natriuretic peptide; NYHA, New York Heart Association; RHR, rest heart rate; SBP, systolic blood pressure.

In HFpEF‐1 patients, older age, HHF in the previous 1 year, NYHA III–IV, NTproBNP ≥1600 pg/mL, DM, AF, COPD, CKD, anemia, SBP less than 120 mmHg, and serum sodium less than 135 mmol/L were independently associated with the occurrence of the primary endpoint in multivariate analysis.

In HFpEF‐2 patients, HHF in the previous 1 year and CKD were independently associated with the occurrence of the primary endpoint.

In HFpEF‐3 patients, DM and SBP less than 120 mmHg were independently associated with the occurrence of the primary endpoint.

In HFpEF‐4 patients, older age, HHF in the previous 1 year NT‐proBNP ≥1600 pg/mL, SBP less than 120 mmHg, and serum sodium greater than 145 mmol/L were independently associated with the occurrence of the primary endpoint.

In HFpEF‐5 patients, HHF in the previous 1 year and NT‐proBNP ≥1600 pg/mL were independently associated with the occurrence of the primary endpoint (Table S7 and Table 4).

## 3 Discussion

This study elucidated distinct clinical characteristics and diverse clinical outcomes among HFpEF patients with different phenotypes, indicating the close association between outcomes and phenotypes among HFpEF patients. Additionally, risk factors for adverse outcomes among patients with different phenotypes were explored; the results showed that risk factors for outcomes varied in HFpEF patients with different phenotypes. To our knowledge, this study represents the first large sample size, nationwide, multicenter, and long‐term follow‐up investigation of clinical characteristics and outcomes among patients with distinct phenotypes of HFpEF.

The implication of this study was as follows: First, patients with different phenotypes of HFpEF exhibited distinct clinical characteristics and outcomes. Recent studies indicate that semaglutide protects against clinical heart failure events in patients with HFpEF, particularly those with the obesity‐related phenotype [20]. Our findings further indicated that HFpEF might be a highly heterogeneous clinical syndrome. The treatment strategy guided by phenotypes might be a promising future therapeutic strategy. Second, risk factors associated with adverse events varied in HFpEF patients with different phenotypes. Patients might benefit more significantly from an optimized therapeutic target for HFpEF patients with different phenotypes. Patients might benefit more significantly from optimized therapeutic targets for HFpEF patients with different phenotypes based on this analysis. Future studies are needed to test this hypothesis.

Several nationwide studies have reported clinical outcomes in HFpEF patients. The ASIAN‐HF study showed 12.1% of patients died or were rehospitalized for HF within 1 year [21]. And the ESC‐HF‐LT studies demonstrated that the rates of all‐cause death, all‐cause hospitalization, and heart failure (HF) hospitalization were 6.3%, 23.5%, and 9.7%, respectively [22]. The study based on the CCA database showed the 1‐year rate of HHF was 13.6% and CV death was 3.1% in HFpEF patients [23]. In our study, the combined endpoint of heart failure hospitalization or cardiovascular death within 1 year after discharge in patients with HFpEF was 10.6%. Approximately 2.5% patients suffered cardiovascular‐related death, while 8.3% of patients experienced HF hospitalization. Additionally, the all‐cause mortality rate was 6.5%, and the all‐cause hospitalization rate was 10.3%. The lower hospitalization rate observed in our study could be attributed primarily to the selection of different endpoints for evaluation. We primarily focused on the occurrence of the heart failure‐related hospitalization event. The observed all‐cause mortality and CV‐related mortality rates in our study were generally consistent with previous studies.

In terms of prognosis among different phenotypes, it is observed that HFpEF‐3, HFpEF‐4, and HFpEF‐5 exhibited worse outcomes compared to HFpEF‐1 and HFpEF‐2. However, within the higher risk phenotypes (‐3, ‐4, ‐5), there were also variations. Specifically, HFpEF‐5 is characterized by a higher mortality rate, whereas HFpEF‐3 and HFpEF‐4 are characterized by elevated re‐admission rates. For HFpEF‐3 and HFpEF‐4, it is necessary to focus on managing clinical symptoms to decrease the rate of rehospitalization. This involves addressing symptoms, optimizing treatment plans, and ensuring thorough follow‐up care.

### 3.1 HFpEF‐1

While HFpEF‐1 and HFpEF‐2 were linked to lower rates of primary endpoints, notable features were also observed. HFpEF‐1, representing vascular‐related HFpEF, encompasses cases associated with hypertension, coronary artery disease (CAD), and coronary microvascular dysfunction. This phenotype was identified as the most prevalent in our study and linked with better clinical outcomes compared to other phenotypes. Hypertension and CAD were recognized as the most prevalent cardiovascular comorbidities in HFpEF [24, 25]. Additionally, coronary microvascular dysfunction emerged as a common pathophysiological mechanism in HFpEF [24, 26]. Within this phenotype, hypertension, ischemia resulting from epicardial CAD, or microvascular dysfunction were pivotal factors in the pathophysiology of HFpEF. Theoretically, these patients might benefit from therapeutic strategies employed in HFrEF. Our study revealed that this particular phenotype had a higher proportion of ACEI/ARB utilization in clinical practice. It might partially explain the lower incidence of both primary and secondary endpoints in HFpEF‐1. Age, prior HHF history, NYHA stage, NT‐proBNP level, Diabetes, AF, CKD, and anemia are well‐established prognostic factors, as we found in HFpEF‐1 patients in the present study. Recently, COPD has emerged as the prominent predictor for all‐cause and cardiovascular mortality in HFpEF [27], and it may contribute partly to the adverse events associated with HFpEF [28]. Therefore, it is essential to conduct further investigations to elucidate the specific underlying mechanisms. An independent association between serum sodium level less than 135 mmol/L and adverse clinical outcomes has been consistently observed, as reported in previous studies [29, 30]. These findings underscore the significance of maintaining serum sodium levels within the normal range. We also found that SBP less than 120 mmHg was associated with adverse events in HFpEF‐1 patients. The PARAGON‐HF trial demonstrated that HFpEF patients who had baseline and mean achieved SBP levels ranging from 120 to 129 mmHg exhibited the lowest risk of experiencing adverse clinical outcomes. Previous studies also indicated the presence of a U‐shaped relationship between baseline SBP and clinical outcomes [23, 31]. This suggested that more precise blood pressure control is necessary for patients with HFpEF‐1 to optimize clinical outcomes.

### 3.2 HFpEF‐2

HFpEF‐2 patients exhibited conditions such as hypertrophic cardiomyopathy, infiltrative cardiomyopathies like cardiac amyloidosis and Fabry cardiomyopathy, and so forth. This group demonstrated better clinical outcomes compared to other groups. Diastolic dysfunction and elevated left ventricular filling pressure were considered the underlying pathophysiology in these patients. Hypertrophic cardiomyopathy (HCM) and restrictive cardiomyopathy (RCM) were identified as the main types of cardiomyopathy‐related HFpEF [32]. Systolic dysfunction resulting from adverse left ventricular remodeling was a significant contributor to the progression and mortality of HF [33, 34, 35]. Hence, LVEF served as a crucial indicator for assessing the severity of HF in these patients. The indication of preserved ejection fraction often suggests an early stage of the disease. Consequently, in our study, these patients exhibited a younger age and larger LVEDD. The incidence of primary and secondary endpoints at the 1‐year follow‐up was lower. This may be attributable, at least partially, to the early phase of the primary disease and the elevated incidence of implantable devices.

### 3.3 HFpEF‐3

HFpEF‐3 represented right heart‐ and pulmonary‐related HFpEF. Patients within this subtype commonly exhibited pulmonary hypertension, with or without right ventricular dysfunction. Importantly, pulmonary hypertension and right ventricular dysfunction could arise due to elevated left ventricular filling pressure and left atrial hypertension, thereby contributing to the development of HFpEF. Previous studies have highlighted that right ventricular dysfunction (RVD) was highly prevalent in HFpEF patients and was associated with a poor prognosis [36, 37]. Our observations indicated that the history of DM was independently associated with the occurrence of the primary endpoint. A cross‐sectional study with a small sample size revealed that the presence of DM independently correlated with RV systolic dysfunction. The association between DM and HFpEF‐3 necessitated further investigation and study [38]. Consequently, our findings suggested that effective DM management could play a crucial role in decreasing hospitalization rates for HFpEF‐3 patients. However, it should be noted that while approximately one‐fifth of patients with HFpEF are estimated to have right heart failure or pulmonary hypertension, relatively few HFpEF cases are primarily attributed to isolated right heart disease or pulmonary arterial hypertension (such as idiopathic pulmonary arterial hypertension) [36, 39, 40]. The relatively small number of participants classified as HFpEF‐3 limited the power of our analysis for this specific phenotype, leading to wider confidence intervals in the statistical outcomes. The prognostic value of the HFpEF‐3 classification, therefore, warrants investigation in larger, adequately powered studies to enable a more robust statistical analysis.

### 3.4 HFpEF‐4

HFpEF‐4 corresponded to valvular‐ and rhythm‐related HFpEF. Valvular diseases could result in hemodynamic disturbances in the left and/or right heart, affecting filling pressure and diastolic function. Rhythm‐related HFpEF primarily involved HFpEF associated with AF. Patients in this category might potentially derive benefits from interventions such as surgical or interventional valve therapy [41], as well as rhythm control therapy [42]. Considering our results, more attention should be paid to patients who are older, have a history of heart failure hospitalization in the previous year, NT‐pro‐BNP ≥1600 pg/mL, SBP less than 120 mmHg, and serum sodium levels greater than 145 mmol/L.

### 3.5 HFpEF‐5

HFpEF‐5 represented extra‐cardiac disease‐related HFpEF. This phenotype encompassed various extra‐cardiac diseases. This type of HFpEF was frequently secondary to other advanced primary diseases, and thus, the management and control of these primary diseases become paramount in the overall management of these HFpEF patients [43, 44, 45]. As a consequence, our study demonstrated that this phenotype of patients, despite presenting better cardiac function at baseline, exhibited significantly higher rates of cardiovascular death and all‐cause mortality. The markedly elevated mortality observed in Phenotype 5 likely reflects the systemic nature of disease processes—such as frailty, chronic inflammatory disorders, pulmonary disease, renal impairment, or malignancy—that contribute to extracardiac organ dysfunction and biological aging pathways. These findings indicate that in this patient cohort, management of the underlying condition is equally critical as prevention of cardiovascular mortality. Active management of the primary condition should be complemented by close cardiac monitoring—for instance, through ambulatory electrocardiogram enhancement and tracking of echocardiographic parameter dynamics. Optimal care of such patients necessitates a multidisciplinary approach involving cardiologists to ensure integrated therapeutic decision‐making. Prior HHF history and NT‐proBNP level were independently associated with the occurrence of the primary endpoint. Furthermore, these two indicators could also serve as indicators of the severity of systemic disease. In the future management of patients with this phenotype, special attention should be given to these two risk factors. However, due to insufficient granularity of recorded comorbidities in the current dataset, we could not identify which specific conditions predominantly drove the risk. Future phenotype‐guided research incorporating richer multimorbidity profiling—including inflammatory biomarkers, frailty assessments, and detailed organ‐specific diagnoses—will be essential for disentangling the heterogeneous mechanisms underlying HFpEF‐5.

### 3.6 Distinct High‐Risk Profiles Across Phenotypes

The markedly different risk profiles observed among high‐risk phenotypes provide important clinical insight. HFpEF‐3 and HFpEF‐4 exhibited disproportionately high rates of HF re‐admission, likely reflecting their predominant hemodynamic and atrial‐structural vulnerability. In contrast, HFpEF‐5 showed substantially elevated rates of both cardiovascular and non‐cardiovascular mortality. The similar proportions of these two causes of death suggest that excess risk in this phenotype may result not only from HF progression itself but also from the burden and deterioration of systemic comorbidities such as chronic kidney disease, pulmonary disease, frailty, or occult malignancy. Because detailed cause‐of‐death adjudication and comorbidity‐specific trajectories were not available, attribution bias cannot be excluded. This underscores the need for integrated management strategies targeting both HF and multimorbidity, and highlights the importance of future studies with more granular phenotyping and longitudinal disease‐specific outcomes to disentangle the mechanisms underlying the heightened mortality in HFpEF‐5.

### 3.7 Phenotype‐Specific Patterns of Risk Factors

The multivariable analyses showed that certain clinical variables, such as anemia or chronic kidney disease, emerged as independent predictors in some phenotypes but not in others. This pattern should be interpreted in the context of both statistical power and biological heterogeneity. On the one hand, the marked differences in sample size and event rates between phenotypes result in unequal power; in many cases, the direction and approximate magnitude of the hazard ratios were similar across phenotypes, but smaller subgroups yielded wider confidence intervals and non‐significant *p*‐values, particularly outside the largest phenotype. On the other hand, it is also plausible that the prognostic impact of specific comorbidities differs across pathophysiological backgrounds. For example, in comorbidity‐rich phenotypes, anemia and chronic kidney disease may more strongly reflect systemic congestion, inflammation, and multiorgan vulnerability, whereas in more vascular‐dominant or atrial‐dominant phenotypes other factors may play a relatively greater role. These findings are consistent with previous reports highlighting phenotype‐dependent prognostic markers in HFpEF and should be considered hypothesis‐generating, warranting confirmation in larger cohorts with formal interaction testing.

### 3.8 Strengths and Limitations

Our study possessed several strengths, including the large sample size and nationwide real‐world nature, representative of contemporary HFpEF phenotype status and outcome in China. Our study validated that there was distinguished incidence of primary endpoints, such as HF‐related rehospitalization and CV death within 1 year after discharge among HFpEF patients with diverse etiologies. This finding highlights the importance of developing more personalized and effective therapies based on phenotypes to improve the management and outcome of HFpEF patients based on their individual phenotype. There were also some limitations in this study. One of the limitations was the incompleteness of some baseline data on the registry. This is a common challenge that in real‐world studies might generate biased estimates. However, although some covariates had missing values, the overall missingness was low (<10%), and multiple imputation analyses produced findings consistent with the complete‐case results. Therefore, missing data are unlikely to have materially influenced the study conclusions. Second, although the attrition rate was relatively low (6.3%), participants lost to follow‐up exhibited modest differences in several baseline characteristics (SMD >0.10), indicating that loss to follow‐up was not entirely random. To evaluate the potential influence of this imbalance, we conducted a worst‐case and best‐case extreme scenario analysis. The results under both assumptions remained consistent with the main findings, supporting the robustness of our conclusions. Therefore, while some degree of selection bias cannot be completely excluded, its impact on the overall study interpretation is likely minimal. Third, it is worth noting that some HFpEF patients may potentially have more than one phenotype based on their etiological factors. However, as our study focused on the first time of endpoint events in patients with HFpEF, we believe it is crucial to concentrate on the primary etiology leading to heart failure. Therefore, for patients meeting multiple classification criteria, we adopted an approach where clinicians adjudicate the principal cause for classification. It should be noted that the current methodology may not fully capture the complex interplay of comorbid conditions, potentially leading to an underestimation of their synergistic effect on the multifactorial pathogenesis of heart failure. Present study coded all patients with single phenotype, and this issue needs to be considered in future studies. Inter‐rater reliability of phenotype assignment was not formally assessed in the current registry cycle, and Kappa statistics were therefore unavailable. This limitation should be addressed in future studies through standardized training and adjudication procedures. Fourth, an important consideration is that HFpEF phenotypes are not fixed entities; instead, patients may transition between phenotypes as their comorbidities progress or acute cardiovascular events occur. For example, an individual initially assigned to the atrial fibrillation‐related phenotype (HFpEF‐4) may develop an acute myocardial infarction during follow‐up, resulting in substantial hemodynamic deterioration and a phenotype more aligned with the vascular‐dominant subtype (HFpEF‐1). Because our current dataset does not yet include 3–5‐year follow‐up, we were unable to capture or quantify these potential phenotype shifts or assess their long‐term prognostic implications. Ongoing longitudinal data collection within the registry will allow future analyses to characterize phenotype evolution and determine whether dynamic phenotype changes meaningfully modify risk over time. Fourth, it should be noted that few HFpEF cases were primarily attributed to isolated right heart disease or pulmonary arterial hypertension (such as idiopathic pulmonary arterial hypertension) [21, 25, 26]. The small number of participants classified as HFpEF‐3 limited the power of our analysis for this specific phenotype; especially, the results of the multivariate analysis are exploratory and must be interpreted with considerable caution due to the limited sample size. Future studies with larger or pooled cohorts will be necessary to validate the prognostic characteristics of smaller HFpEF phenotypes. Finally, the use of SGLT2 inhibitors was very low in this cohort (2.5%), which primarily reflects the enrollment period when SGLT2 inhibitors had not yet been widely adopted for HF management. As a result, our findings largely represent the treatment patterns of the pre‐SGLT2 inhibitor era. Although the low uptake may limit the generalizability of our results to contemporary HF populations with broader use of SGLT2 inhibitors, the minimal exposure also reduces confounding from differential SGLT2 inhibitor use between phenotypes. Nonetheless, future studies incorporating contemporary guideline‐directed therapies—including SGLT2 inhibitors—are needed to validate whether the observed phenotypic patterns persist in current clinical practice.

## 4 Conclusion

Based on a large sample size and nationwide multicenter clinical data, we demonstrated that our HFpEF phenotyping method provides preliminary evidence of its partial clinical significance in prognostic stratification. Patients with different phenotypes of HFpEF exhibited distinct risks of HF‐related rehospitalization and CV death within 1 year after discharge. These findings established the association between outcome and phenotype among HFpEF patients. A phenotype‐guided approach warrants further investigation to determine its potential to improve HFpEF prognosis.

## 5 Methods

### 5.1 Data Source

Data from the CCA Database‐Heart Failure Center Registry were analyzed. The CCA Database‐Heart Failure Center Registry is a prospective, multicenter, observational study enrolling hospitalized patients with a primary discharge diagnosis of HF in CCA Database‐Heart Failure Center certified secondary or tertiary hospitals of China, which was approved by the Central Ethics Committee of Beijing Hospital (2018BJYYEC‐059‐02). The registry study aimed to formulate uniform accreditation standards for heart failure centers and to examine the clinical profile, management strategies, and prognostic determinants in Chinese patients with HF [23]. Up to December 2023, a total of 853 hospitals had passed certification of the CCA Database‐Heart Failure Center, and this study analyzed patients registered in 610 hospitals certified from 2017 to 2021.

### 5.2 Patients Selection

The diagnostic criteria for HF utilized in this study were in accordance with the Chinese and European HF guidelines [6, 46]. These criteria included the presence of clinical symptoms and/or signs of HF, evidence of cardiac structural and/or functional abnormalities as observed via echocardiographic examination, and elevated levels of serum natriuretic peptide. If LVEF ≥50%, which was derived from echocardiography examination during the index hospitalization, HF was defined as HFpEF [6]. The HFpEF patients were regrouped into five groups by the phenotyping coding proposed before [18] (Table S8). In cases where multiple etiological factors coexisted, investigators were instructed to adjudicate and record the single primary etiology considered to be the dominant driver of the index HF episode, rather than assigning multiple phenotypes.

### 5.3 Data Extraction and Management

The baseline data collected were as follows: demographics (age and sex), anthropometrics (bodyweight, height and body mass index [BMI]), vital signs upon admission, characteristics of HF (precipitating factors for the index HF admission, history of hospitalization for HF in the previous year, clinical symptoms and signs (Quality of Life Questionnaire [QOLQ], Minnesota Heart Failure Quality of Life Scale [MLHFQ], 6‐min walk test [6MWT], and New York Heart Association [NYHA] class), laboratory and echocardiography parameters, comorbidities (such as smoking, alcohol consumption, obesity, hypertension, diabetes mellitus [DM], dyslipidemia, coronary heart disease [CHD], previous myocardial infarction [MI], previous stroke/transient ischemic attack [TIA], peripheral arterial disease [PAD], atrial fibrillation [AF], chronic obstructive pulmonary disease [COPD], chronic kidney disease [CKD], anxiety/depression, and cancer), prior interventional and device therapy, and discharge medication. In this study, obesity was defined as a body mass index (BMI) of ≥28 kg/m^2^ upon discharge [47]. CKD was defined as an estimated glomerular filtration rate (eGFR) of less than 60 mL/min/1.73 m^2^ using the Modification of Diet in Renal Disease formula [48]. Anemia was defined as serum hemoglobin concentration less than 12 g/dL for men and less than 11 g/dL for women [49].

### 5.4 Study Endpoints

The primary endpoint was a composite of rehospitalization due to heart failure (HHF) or cardiovascular (CV) death within 1 year, and the secondary endpoint was all‐cause death and all‐cause hospitalization within 1 year after discharge.

### 5.5 Statistical Analysis

For continuous variables, means with standard deviations were reported for normally distributed data, while medians with interquartile ranges were reported for non‐normally distributed data. Comparisons between groups were performed using one‐way ANOVA or Kruskal–Wallis tests, depending on the distribution of the data. Categorical variables were presented as counts with percentages and compared using chi‐squared tests or Fisher's exact tests when appropriate.

Clinical outcomes were expressed as event rate at 1‐year follow‐up and number of events per 100 person‐years, respectively. Time‐to‐first event for the primary composite endpoint (first HHF or CV death) was compared across the five HFpEF phenotypes using Kaplan–Meier curves with log‐rank tests and Cox proportional hazards models with the HFpEF‐2 group as the reference.

For single endpoints, Fine–Gray subdistribution hazard models were used, with death or non‐CV death considered as competing events.

All analyses of the primary composite endpoint and the individual endpoints were adjusted for established prognostic predictors of HFpEF reported in previous literature [23], including age, systolic blood pressure less than 120 mmHg or greater than 130 mmHg, resting heart rate ≥60 bpm, prior hospitalization for heart failure within 12 months, NYHA Class III–IV, LVEF ≥60%, serum sodium less than 135 mmol/L or greater than 145 mmol/L, serum potassium more than 5.5 mmol/L, NT‐proBNP ≥1600 pg/mL, and COPD.

Proportional hazards assumptions were examined (e.g., Schoenfeld residuals) and addressed as needed. To evaluate factors associated with the primary endpoint, univariate and multivariable Cox proportional hazard analyses were performed. Factors were prespecified based on clinical relevance, and those with *p *< 0.1 in univariate analysis were subsequently included in the multivariable model.

### 5.6 Sensitivity Analysis for Loss to Follow‐Up

To assess the potential impact of attrition on the robustness of the findings, we performed a worst‐case and best‐case extreme scenario analysis. For the worst‐case scenario, all participants lost to follow‐up were assumed to have experienced the adverse outcome of interest. For the best‐case scenario, all lost participants were assumed to have remained event‐free throughout follow‐up. The primary endpoint analyses (Cox/logistic models) were re‐estimated under both assumptions to evaluate whether the direction or magnitude of associations differed meaningfully from the main results.

### 5.7 Missing Data Handling

Given the large overall sample size and the relatively low proportion of missing values for covariates included in the multivariable models (all <10%), the primary analyses were conducted using a complete‐case approach. To assess the robustness of the findings to different assumptions about missingness, we additionally performed multiple imputation using chained equations (MICE) as a sensitivity analysis. Twenty imputed datasets were generated including all variables used in the outcome models, and regression estimates were pooled using Rubin's rules.

All data were analyzed using R software (version 4.2.1, R Foundation), and *p *< 0.05 was considered statistically significant.

## Author Contributions

Shuai Yuan, Zhonglei Xie, Xiaotong Cui, Jiefu Yang, Jingmin Zhou, and Junbo Ge designed the research and drafted the manuscript. Yanyan Wang, Shun Yao, Yugang Dong, Yuhua Liao, Weimin Li, and Xinli Li presided over the enrollment and exclusion of patients and collected the data. Xiaotong Cui, Yanyan Wang, Yugang Dong, Yuhua Liao, Weimin Li, and Xinli Li checked the data. Shuai Yuan, Zhonglei Xie, and Yamei Xu statistically analyzed the data. Kai Hu, Jiefu Yang, Jingmin Zhou, and Junbo Ge supervised the conduction of the study. Xiaotong Cui, Kai Hu, Jiefu Yang, Jingmin Zhou, and Junbo Ge revised the manuscript. All authors have read and approved the final manuscript.

## Funding

Our study was supported by the funding of National Key R&D Program of China (Grant Number: 2018YFE0103000), National Natural Science Foundation of China (Grant Numbers: 82200423, 82300435, 82500462), and Shanghai Top Priority research center construction project (2022ZZ01010).

## Ethics Statement

The study was approved by the Central Ethics Committee of Beijing Hospital (2018BJYYEC‐059‐02).

## Conflicts of Interest

The authors declare no conflicts of interest.

## Supporting information




**TABLE S1**. Baseline clinical characteristics of included and excluded cohorts. **TABLE S2**. Baseline clinical characteristics of study cohort and lack of follow‐up data cohort. **TABLE S3**. Primary endpoint at 1‐year follow‐up in the five phenotypes in worst‐case scenario.**TABLE S4**. Primary endpoint at 1‐year follow‐up in the five phenotypes in best‐case scenario. **TABLE S5**. The proportion of missing values for covariates included in the multivariable models. **TABLE S6**. Clinical outcome at 1‐year follow‐up in the five phenotypes under multiple imputation analyses.**TABLE S7**. Factors associated with primary endpoint by different HFpEF phenotypes in univariate Cox model at 1‐year follow‐up. **TABLE S8**. Operational definitions and prioritization principles for HFpEF phenotypes.

## Data Availability

The data that support the findings of this study are available from the corresponding author upon reasonable request.
