# Supplementary material for: Phenotypes and Clinical Outcome of Heart Failure With Preserved Ejection Fraction Patients in China: Findings From the Chinese Cardiovascular Association Database‐Heart Failure Center Registry
Source: MedComm (2020). 2026 Feb 19;7(3):e70642. doi: 10.1002/mco2.70642 (PMC12921267; doi:10.1002/mco2.70642)
Supplement: Supplementary file 1 — TABLE S1. Baseline clinical characteristics of included and excluded cohorts. TABLE S2. Baseline clinical characteristics of study cohort and lack of follow‐up data cohort. TABLE S3. Primary endpoint at 1‐year follow‐up in the five phenotypes in worst‐case scenario.TABLE S4. Primary endpoint at 1‐year follow‐up in the five phenotypes in best‐case scenario. TABLE S5. The proportion of missing values for covariates included in the multivariable models. TABLE S6. Clinical outcome at 1‐year follow‐up in the five phenotypes under multiple imputation analyses.TABLE S7. Factors associated with primary endpoint by different HFpEF phenotypes in univariate Cox model at 1‐year follow‐up. TABLE S8. Operational definitions and prioritization principles for HFpEF phenotypes. [file MCO2-7-e70642-s001.docx]

**SUPPLEMENTAL MATERIAL**

**Phenotypes and clinical outcome of heart failure with preserved ejection fraction (HFpEF) patients in China: Findings from the Chinese Cardiovascular Association Database-Heart Failure Center Registry**

Shuai Yuan, PhD^1#^; Zhonglei Xie, MD^1,2#^; Xiaotong Cui, MD^1#^; Shun Yao, PhD^1#^; Yamei Xu, MD^1^; Yanyan Wang, PhD^1^; Kai Hu, MD, PhD^1^; Yugang Dong, MD^3^; Yuhua Liao, MD^4^; Weimin Li, MD^5^; Xinli Li, MD^6^; Jiefu Yang, MD^7*^; Jingmin Zhou, MD^1*^, Junbo Ge, MD^1*^

1. Department of Cardiology, Zhongshan Hospital Fudan University, Shanghai Institute of Cardiovascular Diseases, Shanghai, China;
2. Institutes of Biomedical Sciences Fudan University, Shanghai, China;
3. Department of Cardiology, the First Affiliated Hospital of Sun Yat-sen University, Guangzhou, China;
4. Department of Cardiology, Union Hospital, Tongji Medical College, Huazhong University of Science and Technology, Wuhan, China;
5. Department of Cardiology, the First Affiliated Hospital of Harbin Medical University, Harbin, China;
6. Department of Cardiology, the First Affiliated Hospital of Nanjing Medical University, Nanjing, China;
7. Department of Cardiology, Beijing Hospital, Beijing, China

* These authors contribute equally to the work.

**#Correspondence to:**

Junbo Ge, MD, Department of Cardiology, Zhongshan Hospital Fudan University, Shanghai Institute of Cardiovascular Diseases, 180 Fenglin Road, Xuhui District, Shanghai, 200032, China.

E‐mail: [jbge@zs-hospital.sh.cn](mailto:jbge@zs-hospital.sh.cn);

Jingmin Zhou, MD, Department of Cardiology, Zhongshan Hospital Fudan University, Shanghai Institute of Cardiovascular Diseases, 180 Fenglin Road, Xuhui District, Shanghai, 200032, China.

E‐mail: zhou.jingmin@zs-hospital.sh.cn;

Jiefu Yang, MD, Department of Cardiology, Beijing Hospital, No.1 Dahua Dongdan Road, Dongcheng District, Beijing, 100730, China.

E-mail: [yangjiefu2011@126.com](mailto:yangjiefu2011@126.com)

**This file includes:**

Supplemental Table S1-7.

| **Table S1 Baseline clinical characteristics of included and excluded cohorts** | | | | | |
| --- | --- | --- | --- | --- | --- |
|  |  | **Overall** | **Excluded** | **Included** | **SMD** |
| n (%) |  | 124,536 | 73070 (58.7%) | 51466 (41.3%) |  |
| Age (median [IQR]) | | 74.00 [66.00, 82.00] | 74.00 [65.00, 82.00] | 74.00 [66.00, 82.00] | 0.036 |
| Sex (%) | Female | 62417 (50.1) | 36791 (50.4) | 25626 (49.8) | 0.011 |
|  | Male | 62070 (49.8) | 36252 (49.6) | 25818 (50.2) |  |
|  | Unknown | 49 (0.0) | 27 (0.0) | 22 (0.0) |  |
| Occupation (%) | Agricultural, manufacturing, services or sales workers | 32416 (26.0) | 19916 (27.3) | 12500 (24.3) | 0.095 |
|  | Housework, retired, unemployed or other occupations | 82653 (66.4) | 48110 (65.8) | 34543 (67.1) |  |
|  | Managers or professionals | 1846 (1.5) | 1121 (1.5) | 725 (1.4) |  |
|  | Unknown | 7621 (6.1) | 3923 (5.4) | 3698 (7.2) |  |
| Alcohol consumption(%) | Never | 99246 (79.7) | 58056 (79.5) | 41190 (80.0) | 0.028 |
|  | Previous or current | 21886 (17.6) | 13104 (17.9) | 8782 (17.1) |  |
|  | Unknown | 3404 (2.7) | 1910 (2.6) | 1494 (2.9) |  |
| Previous or current smoker (%) | Never | 88484 (71.1) | 52140 (71.4) | 36344 (70.6) | 0.016 |
|  | Previous or current | 32671 (26.2) | 18959 (25.9) | 13712 (26.6) |  |
|  | Unknown | 3381 (2.7) | 1971 (2.7) | 1410 (2.7) |  |
| BMI (median [IQR]) | | 23.63 [21.22, 26.22] | 23.53 [21.13, 26.12] | 23.74 [21.26, 26.35] | 0.011 |
| SBP (median [IQR]) | | 128.00 [116.00, 141.00] | 127.00 [115.00, 140.00] | 129.00 [116.00, 143.00] | 0.067 |
| DBP (median [IQR]) | | 74.00 [67.00, 81.00] | 74.00 [67.00, 80.00] | 74.00 [66.00, 82.00] | 0.021 |
| RHR (median [IQR]) | | 80.00 [69.00, 92.00] | 80.00 [69.00, 93.00] | 79.00 [68.00, 92.00] | 0.01 |
| HHF in previous 12months (%) | | 20297 (16.3) | 11164 (15.3) | 9133 (17.7) | 0.069 |
| HF_main_inducement (%) | Arrhythmia | 13296 (10.7) | 8311 (11.4) | 4985 (9.7) | 0.096 |
|  | Coronary artyery disease | 34484 (27.7) | 19294 (26.4) | 15190 (29.5) |  |
|  | Infection | 17593 (14.1) | 10316 (14.1) | 7277 (14.1) |  |
|  | Other | 25578 (20.5) | 15519 (21.2) | 10059 (19.5) |  |
|  | Poor adherence to medication | 4938 (4.0) | 2978 (4.1) | 1960 (3.8) |  |
|  | Uncontrolled hypertension | 7352 (5.9) | 4047 (5.5) | 3305 (6.4) |  |
|  | Volume overload | 1565 (1.3) | 913 (1.2) | 652 (1.3) |  |
|  | Unknown | 19730 (15.8) | 11692 (16.0) | 8038 (15.6) |  |
| NYHA (%) | III-IV | 51378 (41.3) | 28906 (39.6) | 22472 (43.7) | 0.084 |
| Glucose (median [IQR]) | | 5.64 [4.90, 7.01] | 5.60 [4.88, 6.96] | 5.70 [4.93, 7.11] | 0.009 |
| Glycosylated hemoglobin (median [IQR]) | | 6.12 [5.70, 7.10] | 6.10 [5.70, 7.10] | 6.18 [5.69, 7.10] | 0.005 |
| Hemoglobin (median [IQR]) | | 129.00 [112.00, 150.00] | 130.00 [112.00, 153.00] | 128.00 [111.00, 148.00] | 0.044 |
| UA (median [IQR]) | | 382.00 [302.00, 481.00] | 380.00 [300.00, 478.00] | 386.00 [305.00, 486.48] | 0.031 |
| Creatinine (median [IQR]) | | 83.00 [66.40, 110.00] | 82.00 [66.00, 108.00] | 84.15 [67.40, 112.00] | 0.023 |
| eGFR (median [IQR]) | | 79.41 [55.69, 102.97] | 80.49 [56.90, 104.33] | 77.89 [54.16, 101.02] | 0.009 |
| BNP (median [IQR]) | | 341.49 [133.00, 884.50] | 329.92 [128.68, 862.00] | 358.00 [140.00, 921.69] | 0.003 |
| NTproBNP (median [IQR]) | | 1,589.96 [599.00, 3,880.72] | 1,587.00 [591.00, 3,891.00] | 1,593.00 [607.48, 3,865.00] | 0.007 |
| Sodium (median [IQR]) | | 140.00 [137.60, 142.00] | 140.00 [137.70, 142.00] | 140.00 [137.50, 142.00] | 0.009 |
| Potassium (median [IQR]) | | 4.05 [3.76, 4.40] | 4.04 [3.75, 4.38] | 4.07 [3.77, 4.40] | <0.001 |
| QOLQ (median [IQR]) | | 70.00 [56.00, 80.00] | 70.00 [58.00, 80.00] | 70.00 [55.00, 80.00] | 0.033 |
| MLHF (median [IQR]) | | 40.00 [20.00, 68.00] | 40.00 [20.00, 68.00] | 40.00 [20.00, 68.00] | 0.009 |
| LVEF (median [IQR]) | | 59.00 [55.00, 64.00] | 59.55 [55.00, 64.00] | 59.00 [55.00, 64.00] | 0.055 |
| LVEDD (median [IQR]) | | 47.00 [43.00, 52.00] | 47.00 [43.00, 52.00] | 48.00 [43.00, 52.00] | 0.014 |
| 6-minute walk distance (median [IQR]) | | 360.00 [263.00, 450.00] | 365.00 [265.00, 454.02] | 350.00 [261.50, 430.00] | 0.02 |
| Obesity (%) |  | 11831 (9.5) | 6681 (9.1) | 5150 (10.0) | 0.034 |
| Hypertension (%) | | 81492 (65.4) | 46266 (63.3) | 35226 (68.4) | 0.109 |
| DM (%) |  | 34543 (27.7) | 19325 (26.4) | 15218 (29.6) | 0.073 |
| MI (%) |  | 24378 (19.6) | 13159 (18.0) | 11219 (21.8) | 0.097 |
| CHD (%) |  | 49158 (39.5) | 28178 (38.6) | 20980 (40.8) | 0.047 |
| AF (%) |  | 49582 (39.8) | 29034 (39.7) | 20548 (39.9) | 0.01 |
| Stroke/TIA (%) |  | 19959 (16.0) | 11284 (15.4) | 8675 (16.9) | 0.04 |
| PAD (%) |  | 13133 (10.5) | 7225 (9.9) | 5908 (11.5) | 0.052 |
| Dyslipidemia (%) | | 19884 (16.0) | 11520 (15.8) | 8364 (16.3) | 0.022 |
| COPD (%) |  | 12795 (10.3) | 7598 (10.4) | 5197 (10.1) | 0.018 |
| OSAHS (%) |  | 457 (0.4) | 258 (0.4) | 199 (0.4) | 0.076 |
| CKD (%) |  | 15825 (12.7) | 8633 (11.8) | 7192 (14.0) | 0.066 |
| Anaemia (%) |  | 32346 (26.0) | 18342 (25.1) | 14004 (27.2) | 0.049 |
| Anxiety (%) |  | 1161 (0.9) | 551 (0.8) | 610 (1.2) | 0.096 |
| Depression (%) |  | 1001 (0.8) | 442 (0.6) | 559 (1.1) | 0.103 |
| Malignant tumor (%) | | 3633 (2.9) | 2108 (2.9) | 1525 (3.0) | 0.012 |
| Thyroid_disease (%) | | 7037 (5.7) | 4032 (5.5) | 3005 (5.8) | 0.02 |
| CRT (%) |  | 457 (0.4) | 272 (0.4) | 185 (0.4) | 0.014 |
| ICD (%) |  | 423 (0.3) | 274 (0.4) | 149 (0.3) | 0.021 |
| Pacemaker (%) |  | 5451 (4.4) | 3173 (4.3) | 2278 (4.4) | 0.012 |
| PCI (%) |  | 17223 (13.8) | 8646 (11.8) | 8577 (16.7) | 0.139 |
| CABG (%) |  | 1144 (0.9) | 593 (0.8) | 551 (1.1) | 0.027 |
| ACEI (%) |  | 31189 (25.0) | 17840 (24.4) | 13349 (25.9) | 0.035 |
| ARB (%) |  | 34705 (27.9) | 19568 (26.8) | 15137 (29.4) | 0.059 |
| ARNI (%) |  | 17617 (14.1) | 10737 (14.7) | 6880 (13.4) | 0.038 |
| Beta blocker (%) | | 88513 (71.1) | 51495 (70.5) | 37018 (71.9) | 0.036 |
| MRA (%) |  | 76821 (61.7) | 44706 (61.2) | 32115 (62.4) | 0.025 |
| SGLT2i (%) |  | 3765 (3.0) | 2456 (3.4) | 1309 (2.5) | 0.048 |
| Diuretics (%) |  | 84307 (67.7) | 49027 (67.1) | 35280 (68.6) | 0.043 |
| Digoxin (%) |  | 16329 (13.1) | 9686 (13.3) | 6643 (12.9) | 0.012 |
| Ivabradine (%) |  | 1630 (1.3) | 872 (1.2) | 758 (1.5) | 0.025 |
| Antiarrhythmics (%) | | 9750 (7.8) | 5746 (7.9) | 4004 (7.8) | 0.067 |
| Antiplatelets (%) | | 71327 (57.3) | 40452 (55.4) | 30875 (60.0) | 0.11 |
| Statin (%) |  | 88048 (70.7) | 50320 (68.9) | 37728 (73.3) | 0.108 |
| Oral anticoagulants (%) | | 37161 (29.8) | 21988 (30.1) | 15173 (29.5) | 0.016 |
| CCB (%) |  | 27176 (21.8) | 15102 (20.7) | 12074 (23.5) | 0.076 |
| Nitrate (%) |  | 37052 (29.8) | 21526 (29.5) | 15526 (30.2) | 0.02 |
| Oral antidiabetics (%) | | 29753 (23.9) | 17040 (23.3) | 12713 (24.7) | 0.035 |
| Inotropes (%) |  | 14994 (12.0) | 8584 (11.7) | 6410 (12.5) | 0.022 |
| BMI, body mass index; SBP, systolic blood pressure; DBP, diastolic blood pressure; RHR:rest heart rate; HHF in previous 12months, heart failure hospitalization in previous 12 months; HF, heart failure; NYHA, New York Heart Assiciation; UA, Uric Acid; eGFR, estimated glomerular filtration rate; NT-proBNP, N-terminal pro-B-type natriuretic peptide; QOLQ, Quality of Life Questionaire; MLHF, Minnesota Living With Heart Failure; LVEF, left ventricular ejection fraction; LVEDD, left ventricular end-diastolic dimension; DM, diabete mellitus; MI, myocardial infarction; CHD, coronary heart disease; AF, atrial fibrillation; TIA,transient ischemic attacks; PAD, peripheral arterial disease; COPD, chronic obstructive pulmonary disease; OSAHS, obstructive sleep apnea-hypopnea syndrome; CKD,chronic kidney disease; CRT, cardiac resynchronization therapy; ICD, implantable cardioverter defibrillator; PCI, percutaneous coronary intervention; CABG, coronary artery bypass grafting; ACEI, angiotensin-converting enzyme inhibitor; ARB, angiotensin receptor blocker; ARNI, angiotensin receptor–neprilysin inhibitor; MRA, mineralocorticoid receptor antagonist; SGLT2i, sodium–glucose cotransporter 2 inhibitor. | | | | | |

| **Table S2 Baseline clinical characteristics of study cohort and lack of follow-up data cohort** | | | | | |
| --- | --- | --- | --- | --- | --- |
|  |  | **Overall** | **Study cohort** | **Lack of follow-up data cohort** | **SMD** |
| n (%) |  | 59,622 | 51466(86.3%) | 8156(13.7%) |  |
| Age (median [IQR]) | | 74.00 [66.00, 82.00] | 74.00 [66.00, 82.00] | 75.00 [67.00, 82.00] | 0.072 |
| Sex (%) | Female | 29802 (50.0) | 25626 (49.8) | 4176 (51.2) | 0.03 |
|  | Male | 29793 (50.0) | 25818 (50.2) | 3975 (48.7) |  |
|  | Unknown | 27 (0.0) | 22 (0.0) | 5 (0.1) |  |
| Occupation (%) | Agricultural, manufacturing, services or sales workers | 14134 (23.7) | 12500 (24.3) | 1634 (20.0) | 0.106 |
|  | Housework, retired, unemployed or other occupations | 40355 (67.7) | 34543 (67.1) | 5812 (71.3) |  |
|  | Managers or professionals | 822 (1.4) | 725 (1.4) | 97 (1.2) |  |
|  | Unknown | 4311 (7.2) | 3698 (7.2) | 613 (7.5) |  |
| Alcohol consumption (%) | Previous or current | 10396 (17.4) | 8782 (17.1) | 1614 (19.8) | 0.086 |
| Previous or current smoker (%) | Previous or current | 15865 (26.6) | 13712 (26.6) | 2153 (26.4) | 0.113 |
| BMI (median [IQR]) | | 23.73 [21.23, 26.30] | 23.74 [21.26, 26.35] | 23.62 [21.09, 26.13] | 0.037 |
| SBP (median [IQR]) | | 130.00 [116.00, 144.00] | 129.00 [116.00, 143.00] | 130.00 [117.00, 147.00] | 0.073 |
| DBP (median [IQR]) | | 74.00 [66.00, 82.00] | 74.00 [66.00, 82.00] | 75.00 [67.00, 84.00] | 0.046 |
| RHR (median [IQR]) | | 79.00 [68.00, 92.00] | 79.00 [68.00, 92.00] | 78.00 [68.00, 92.00] | 0.012 |
| HHF in previous 12months (%) | | 10547 (17.7) | 9133 (17.7) | 1414 (17.3) | 0.25 |
| HF main inducement (%) | Arrhythmia | 5582 (9.4) | 4985 (9.7) | 597 (7.3) | 0.292 |
|  | Coronary artyery disease | 17123 (28.7) | 15190 (29.5) | 1933 (23.7) |  |
|  | Infection | 8345 (14.0) | 7277 (14.1) | 1068 (13.1) |  |
|  | Other | 11752 (19.7) | 10059 (19.5) | 1693 (20.8) |  |
|  | Poor adherence to medication | 2166 (3.6) | 1960 (3.8) | 206 (2.5) |  |
|  | Uncontrolled hypertension | 3749 (6.3) | 3305 (6.4) | 444 (5.4) |  |
|  | Volume overload | 723 (1.2) | 652 (1.3) | 71 (0.9) |  |
|  | Unknown | 10182 (17.1) | 8038 (15.6) | 2144 (26.3) |  |
| NYHA (%) | III-IV | 26175 (43.9) | 22472 (43.7) | 3703 (45.4) | 0.348 |
| Glucose (median [IQR]) | | 5.69 [4.92, 7.10] | 5.70 [4.93, 7.11] | 5.60 [4.85, 6.96] | 0.015 |
| Glycosylated hemoglobin (median [IQR]) | | 6.18 [5.70, 7.10] | 6.18 [5.69, 7.10] | 6.10 [5.80, 7.00] | 0.048 |
| Hemoglobin (median [IQR]) | | 128.00 [110.00, 147.00] | 128.00 [111.00, 148.00] | 126.00 [109.00, 143.85] | 0.094 |
| UA (median [IQR]) |  | 385.10 [305.00, 486.00] | 386.00 [305.00, 486.48] | 380.00 [303.00, 482.18] | 0.061 |
| Creatinine (median [IQR]) | | 84.00 [67.20, 112.00] | 84.15 [67.40, 112.00] | 83.70 [66.90, 114.00] | 0.004 |
| eGFR (median [IQR]) | | 77.87 [54.02, 101.18] | 77.89 [54.16, 101.02] | 77.68 [52.73, 102.49] | 0.01 |
| NTproBNP (median [IQR]) | | 1,610.00 [608.00, 3,947.66] | 1,593.00 [607.48, 3,865.00] | 1,759.00 [612.03, 4,570.00] | 0.043 |
| Sodium (median [IQR]) | | 140.00 [137.50, 142.00] | 140.00 [137.50, 142.00] | 140.20 [137.80, 142.70] | 0.01 |
| Potassium (median [IQR]) | | 4.07 [3.76, 4.40] | 4.07 [3.77, 4.40] | 4.04 [3.74, 4.40] | 0.004 |
| QOLQ (median [IQR]) | | 70.00 [55.00, 80.00] | 70.00 [55.00, 80.00] | 68.00 [50.00, 80.00] | 0.049 |
| MLHF (median [IQR]) | | 40.00 [20.00, 68.00] | 40.00 [20.00, 68.00] | 31.50 [15.00, 60.00] | 0.151 |
| LVEF (median [IQR]) | | 59.00 [55.00, 64.00] | 59.00 [55.00, 64.00] | 60.00 [55.00, 64.00] | 0.097 |
| LVEDD (median [IQR]) | | 47.90 [43.00, 52.00] | 48.00 [43.00, 52.00] | 47.00 [43.00, 52.00] | 0.018 |
| 6-minute walk distance (median [IQR]) | | 350.00 [263.00, 440.00] | 350.00 [261.50, 430.00] | 400.00 [280.00, 480.00] | 0.02 |
| Obesity (%) |  | 5810 (9.7) | 5150 (10.0) | 660 (8.1) | 0.163 |
| Hypertension (%) |  | 40504 (67.9) | 35226 (68.4) | 5278 (64.7) | 0.081 |
| DM (%) |  | 17488 (29.3) | 15218 (29.6) | 2270 (27.8) | 0.043 |
| MI (%) |  | 12755 (21.4) | 11219 (21.8) | 1536 (18.8) | 0.075 |
| CHD (%) |  | 23389 (39.2) | 20980 (40.8) | 2409 (29.5) | 0.413 |
| AF (%) |  | 23726 (39.8) | 20548 (39.9) | 3178 (39.0) | 0.027 |
| Stroke/TIA (%) |  | 10033 (16.8) | 8675 (16.9) | 1358 (16.7) | 0.035 |
| PAD (%) |  | 6596 (11.1) | 5908 (11.5) | 688 (8.4) | 0.102 |
| Dyslipidemia (%) |  | 9661 (16.2) | 8364 (16.3) | 1297 (15.9) | 0.036 |
| COPD (%) |  | 6144 (10.3) | 5197 (10.1) | 947 (11.6) | 0.052 |
| OSAHS (%) |  | 224 (0.4) | 199 (0.4) | 25 (0.3) | 0.432 |
| CKD (%) |  | 8283 (13.9) | 7192 (14.0) | 1091 (13.4) | 0.036 |
| Anaemia (%) |  | 16340 (27.4) | 14004 (27.2) | 2336 (28.6) | 0.165 |
| Anxiety (%) |  | 656 (1.1) | 610 (1.2) | 46 (0.6) | 0.237 |
| Depression (%) |  | 594 (1.0) | 559 (1.1) | 35 (0.4) | 0.238 |
| Malignant tumor (%) | | 1808 (3.0) | 1525 (3.0) | 283 (3.5) | 0.038 |
| Thyroid disease (%) | | 3487 (5.8) | 3005 (5.8) | 482 (5.9) | 0.026 |
| CRT (%) |  | 212 (0.4) | 185 (0.4) | 27 (0.3) | 0.009 |
| ICD (%) |  | 178 (0.3) | 149 (0.3) | 29 (0.4) | 0.036 |
| Pacemaker (%) |  | 2624 (4.4) | 2278 (4.4) | 346 (4.2) | 0.023 |
| PCI (%) |  | 9817 (16.5) | 8577 (16.7) | 1240 (15.2) | 0.04 |
| CABG (%) |  | 612 (1.0) | 551 (1.1) | 61 (0.7) | 0.034 |
| ACEI (%) |  | 15344 (25.7) | 13349 (25.9) | 1995 (24.5) | 0.034 |
| ARB (%) |  | 17460 (29.3) | 15137 (29.4) | 2323 (28.5) | 0.02 |
| ARNI (%) |  | 7635 (12.8) | 6880 (13.4) | 755 (9.3) | 0.13 |
| Beta blocker (%) |  | 42461 (71.2) | 37018 (71.9) | 5443 (66.7) | 0.141 |
| MRA (%) |  | 36969 (62.0) | 32115 (62.4) | 4854 (59.5) | 0.099 |
| SGLT2i (%) |  | 1571 (2.6) | 1309 (2.5) | 262 (3.2) | 0.04 |
| Diuretics (%) |  | 40693 (68.3) | 35280 (68.6) | 5413 (66.4) | 0.05 |
| Digoxin (%) |  | 7896 (13.2) | 6643 (12.9) | 1253 (15.4) | 0.115 |
| Ivabradine (%) |  | 831 (1.4) | 758 (1.5) | 73 (0.9) | 0.095 |
| Antiarrhythmics (%) | | 4699 (7.9) | 4004 (7.8) | 695 (8.5) | 0.041 |
| Antiplatelets (%) |  | 35685 (59.9) | 30875 (60.0) | 4810 (59.0) | 0.027 |
| Statin (%) |  | 43352 (72.7) | 37728 (73.3) | 5624 (69.0) | 0.097 |
| Oral anticoagulants (%) | | 17567 (29.5) | 15173 (29.5) | 2394 (29.4) | 0.087 |
| CCB (%) |  | 13956 (23.4) | 12074 (23.5) | 1882 (23.1) | 0.073 |
| Nitrate (%) |  | 18561 (31.1) | 15526 (30.2) | 3035 (37.2) | 0.181 |
| Oral antidiabetics (%) | | 14869 (24.9) | 12713 (24.7) | 2156 (26.4) | 0.104 |
| Inotropes (%) |  | 7628 (12.8) | 6410 (12.5) | 1218 (14.9) | 0.106 |
| BMI, body mass index; SBP, systolic blood pressure; DBP, diastolic blood pressure; RHR:rest heart rate; HHF in previous 12months, heart failure hospitalization in previous 12 months; HF, heart failure; NYHA, New York Heart Assiciation; UA, Uric Acid; eGFR, estimated glomerular filtration rate; NT-proBNP, N-terminal pro-B-type natriuretic peptide; QOLQ, Quality of Life Questionaire; MLHF, Minnesota Living With Heart Failure; LVEF, left ventricular ejection fraction; LVEDD, left ventricular end-diastolic dimension; DM, diabete mellitus; MI, myocardial infarction; CHD, coronary heart disease; AF, atrial fibrillation; TIA,transient ischemic attacks; PAD, peripheral arterial disease; COPD, chronic obstructive pulmonary disease; OSAHS, obstructive sleep apnea-hypopnea syndrome; CKD,chronic kidney disease; CRT, cardiac resynchronization therapy; ICD, implantable cardioverter defibrillator; PCI, percutaneous coronary intervention; CABG, coronary artery bypass grafting; ACEI, angiotensin-converting enzyme inhibitor; ARB, angiotensin receptor blocker; ARNI, angiotensin receptor–neprilysin inhibitor; MRA, mineralocorticoid receptor antagonist; SGLT2i, sodium–glucose cotransporter 2 inhibitor. | | | | | |

| **Table S3 Primary endpoint at 1-year follow-up in 5 phenotypes in worst-case scenario.** | | |
| --- | --- | --- |
| **Phenotypes** | **HR (95%CI)** | **P value** |
| Primary endpoint |  |  |
| HFpEF-2 | Reference |  |
| HFpEF-1 | 1.11 (0.9-1.36) | 0.334 |
| HFpEF-3 | 1.71 (1.05-2.77) | 0.03 |
| HFpEF-4 | 1.29 (1.04-1.61) | 0.02 |
| HFpEF-5 | 1.53 (1.12-2.09) | 0.008 |

| **Table S4 Primary endpoint at 1-year follow-up in 5 phenotypes in best-case scenario** | | |
| --- | --- | --- |
| **Phenotypes** | **HR (95%CI)** | **P value** |
| Primary endpoint |  |  |
| HFpEF-2 | Reference |  |
| HFpEF-1 | 1.12 (0.91-1.39) | 0.272 |
| HFpEF-3 | 1.72 (1.06-2.78) | 0.029 |
| HFpEF-4 | 1.31 (1.05-1.63) | 0.017 |
| HFpEF-5 | 1.54 (1.13-2.11) | 0.007 |

| **Table S5 The proportion of missing values for covariates included in the multivariable models** | | |
| --- | --- | --- |
|  |  | **Missing (%)** |
| Age (median [IQR]) | | 0.3 |
| Sex (%) | Female | 0 |
|  | Male |  |
|  | Unknown |  |
| Occupation (%) | Agricultural, manufacturing, services or sales workers | 0 |
|  | Housework, retired, unemployed or other occupations | |
|  | Managers or professionals | |
|  | Unknown |  |
| Alcohol consumption (%) | Previous or current | 0 |
| Previous or current smoker (%) | Previous or current | 0 |
| BMI (median [IQR]) | | 33.2 |
| SBP (median [IQR]) | | 1.6 |
| DBP (median [IQR]) | | 1.6 |
| RHR (median [IQR]) | | 2.7 |
| HHF in previous 12months (%) | | 0 |
| HF main inducement (%) | Arrhythmia | 0 |
|  | Coronary artyery disease | |
|  | Infection | |
|  | Other |  |
|  | Poor adherence to medication | |
|  | Uncontrolled hypertension | |
|  | Unknown |  |
|  | Volume overload | |
| NYHA (%) | III-IV | 0 |
| Glucose (median [IQR]) | | 14.5 |
| Glycosylated hemoglobin (median [IQR]) | | 78.3 |
| Hemoglobin (median [IQR]) | | 5 |
| UA (median [IQR]) | | 58.9 |
| Creatinine (median [IQR]) | | 3.5 |
| eGFR (median [IQR]) | | 3.8 |
| NTproBNP (median [IQR]) | | 5.9 |
| Sodium (median [IQR]) | | 2.9 |
| Potassium (median [IQR]) | | 2.8 |
| QOLQ (median [IQR]) | | 57 |
| MLHF(median [IQR]) | | 85.7 |
| LVEF (median [IQR]) | | 0 |
| LVEDD (median [IQR]) | | 14.3 |
| 6-minute walk distance (median [IQR]) | | 90.2 |
| Obesity (%) |  | 0 |
| Hypertension(%) |  | 0 |
| DM (%) |  | 0 |
| MI (%) |  | 0 |
| CHD (%) |  | 0 |
| AF (%) |  | 0 |
| Stroke/TIA (%) |  | 0 |
| PAD (%) |  | 0 |
| Dyslipidemia (%) | | 0 |
| COPD (%) |  | 0 |
| OSAHS (%) |  | 0 |
| CKD (%) |  | 0 |
| Anaemia (%) |  | 0 |
| Anxiety (%) |  | 0 |
| Depression (%) |  | 0 |
| Malignant tumor (%) | | 0 |
| Thyroid disease (%) | | 0 |
| CRT (%) |  | 0 |
| ICD (%) |  | 0 |
| Pacemaker (%) |  | 0 |
| PCI (%) |  | 0 |
| CABG (%) |  | 0 |
| ACEI (%) |  | 0 |
| ARB (%) |  | 0 |
| ARNI (%) |  | 0 |
| Beta blocker (%) | | 0 |
| MRA (%) |  | 0 |
| SGLT2i (%) |  | 0 |
| Diuretics (%) |  | 0 |
| Digoxin (%) |  | 0 |
| Ivabradine (%) |  | 0 |
| Antiarrhythmics (%) | | 0 |
| Antiplatelets (%) | | 0 |
| Statin (%) |  | 0 |
| Oral anticoagulants (%) | | 0 |
| CCB (%) |  | 0 |
| Nitrate (%) |  | 0 |
| Oral antidiabetics (%) | | 0 |
| Inotropes (%) |  | 0 |
| BMI, body mass index; SBP, systolic blood pressure; DBP, diastolic blood pressure; RHR:rest heart rate; HHF in previous 12months, heart failure hospitalization in previous 12 months; HF, heart failure; NYHA, New York Heart Assiciation; UA, Uric Acid; eGFR, estimated glomerular filtration rate; NT-proBNP, N-terminal pro-B-type natriuretic peptide; QOLQ, Quality of Life Questionaire; MLHF, Minnesota Living With Heart Failure; LVEF, left ventricular ejection fraction; LVEDD, left ventricular end-diastolic dimension; DM, diabete mellitus; MI, myocardial infarction; CHD, coronary heart disease; AF, atrial fibrillation; TIA,transient ischemic attacks; PAD, peripheral arterial disease; COPD, chronic obstructive pulmonary disease; OSAHS, obstructive sleep apnea-hypopnea syndrome; CKD,chronic kidney disease; CRT, cardiac resynchronization therapy; ICD, implantable cardioverter defibrillator; PCI, percutaneous coronary intervention; CABG, coronary artery bypass grafting; ACEI, angiotensin-converting enzyme inhibitor; ARB, angiotensin receptor blocker; ARNI, angiotensin receptor–neprilysin inhibitor; MRA, mineralocorticoid receptor antagonist; SGLT2i, sodium–glucose cotransporter 2 inhibitor. | | |

| **Table S6 Clinical outcome at 1-year follow-up in 5 phenotypes under multiple imputation analyses** | | |
| --- | --- | --- |
| **Phenotypes** | **HR (95%CI)** | **P-value** |
| Primary endpoint |  |  |
| HFpEF-2 | Reference |  |
| HFpEF-1 | 1.11 (0.93-1.33) | 0.259 |
| HFpEF-3 | 1.71 (1.12-2.63) | 0.014 |
| HFpEF-4 | 1.28 (1.06-1.55) | 0.011 |
| HFpEF-5 | 1.39 (1.05-1.84) | 0.022 |
| All-cause death |  |  |
| HFpEF-2 | Reference |  |
| HFpEF-1 | 1.11 (0.88-1.39) | 0.375 |
| HFpEF-3 | 1.67 (0.94-2.95) | 0.078 |
| HFpEF-4 | 1.13 (0.89-1.44) | 0.33 |
| HFpEF-5 | 2.18 (1.61-2.95) | <0.001 |
| CV death |  |  |
| HFpEF-2 | Reference |  |
| HFpEF-1 | 1.13 (0.79-1.62) | 0.5 |
| HFpEF-3 | 1.89 (0.83-4.29) | 0.128 |
| HFpEF-4 | 1.22 (0.84-1.78) | 0.296 |
| HFpEF-5 | 2.84 (1.78-4.52) | <0.001 |
| Non-CV death |  |  |
| HFpEF-2 | Reference |  |
| HFpEF-1 | 0.89 (0.64-1.24) | 0.497 |
| HFpEF-3 | 1.58 (0.67-3.75) | 0.301 |
| HFpEF-4 | 1.03 (0.72-1.46) | 0.878 |
| HFpEF-5 | 2.07 (1.32-3.23) | 0.001 |
| First all-cause hospitalization |  |  |
| HFpEF-2 | Reference |  |
| HFpEF-1 | 1.15 (0.96-1.38) | 0.141 |
| HFpEF-3 | 1.44 (0.9-2.31) | 0.131 |
| HFpEF-4 | 1.24 (1.02-1.51) | 0.029 |
| HFpEF-5 | 0.92 (0.66-1.29) | 0.634 |
| First CV hospitalization |  |  |
| HFpEF-2 | Reference |  |
| HFpEF-1 | 1.16 (0.96-1.42) | 0.13 |
| HFpEF-3 | 1.56 (0.96-2.55) | 0.074 |
| HFpEF-4 | 1.3 (1.06-1.6) | 0.013 |
| HFpEF-5 | 0.87 (0.61-1.25) | 0.459 |
| First HF hospitalization |  |  |
| HFpEF-2 | Reference |  |
| HFpEF-1 | 1.1 (0.9-1.35) | 0.362 |
| HFpEF-3 | 1.61 (0.97-2.67) | 0.063 |
| HFpEF-4 | 1.3 (1.05-1.61) | 0.016 |
| HFpEF-5 | 0.88 (0.61-1.28) | 0.512 |
| CV death, cardiovascular-related death; First HHF, first time of heart failure hospitalization; No-CV death, not cardiovascular-related death; CV hospitalization, cardiovascular related hospitalization | | |

| **Table S7 Factors associated with primary endpoint by different HFpEF phenotypes in univariate cox model at 1-year follow-up** | | | | | | | | | | | | |
| --- | --- | --- | --- | --- | --- | --- | --- | --- | --- | --- | --- | --- |
|  | **Total** |  | **HFpEF-1** |  | **HFpEF-2** |  | **HFpEF-3** |  | **HFpEF-4** |  | **HFpEF-5** |  |
|  | **HR (95%CI)** | **P** | **HR (95%CI)** | **P** | **HR (95%CI)** | **P** | **HR (95%CI)** | **P** | **HR (95%CI)** | **P** | **HR (95%CI)** | **P** |
| Age | 1.02 (1.02-1.02) | <0.001 | 1.02 (1.02-1.03) | <0.001 | 1.01 (1-1.02) | 0.047 | 1.01 (0.98-1.03) | 0.502 | 1.02 (1.01-1.02) | <0.001 | 1.02 (1-1.04) | 0.061 |
| Female | 1.11 (1.05-1.17) | <0.001 | 1.12 (1.05-1.19) | <0.001 | 0.91 (0.67-1.23) | 0.534 | 2.13 (0.86-5.29) | 0.101 | 0.99 (0.88-1.13) | 0.926 | 1.03 (0.66-1.59) | 0.906 |
| HHF in previous 12 months | 1.99 (1.87-2.11) | <0.001 | 1.87 (1.74-2.02) | <0.001 | 2.24 (1.63-3.09) | <0.001 | 0.96 (0.36-2.53) | 0.932 | 2.41 (2.11-2.77) | <0.001 | 2.3 (1.44-3.67) | <0.001 |
| NYHA III-IV | 1.5 (1.41-1.59) | <0.001 | 1.59 (1.49-1.71) | <0.001 | 1.55 (1.13-2.13) | 0.007 | 0.89 (0.4-1.95) | 0.763 | 1.16 (1.01-1.33) | 0.032 | 1.58 (1.01-2.47) | 0.046 |
| LVEF >=60% | 0.89 (0.85-0.94) | <0.001 | 0.89 (0.84-0.95) | <0.001 | 0.65 (0.47-0.9) | 0.009 | 0.66 (0.31-1.41) | 0.287 | 0.96 (0.85-1.09) | 0.568 | 0.91 (0.59-1.4) | 0.656 |
| NT-proBNP >=1600pg/ml | 1.94 (1.82-2.07) | <0.001 | 1.97 (1.83-2.13) | <0.001 | 1.69 (1.17-2.44) | 0.005 | 2.57 (0.94-7.01) | 0.066 | 1.73 (1.49-2.01) | <0.001 | 2.95 (1.7-5.11) | <0.001 |
| RHR >=60bpm | 1.14 (1.03-1.26) | 0.014 | 1.08 (0.96-1.22) | 0.179 | 1.82 (0.93-3.56) | 0.081 | 0.81 (0.19-3.43) | 0.779 | 1.3 (1-1.68) | 0.05 | 1.3 (0.53-3.21) | 0.571 |
| Hypertension | 1.04 (0.98-1.1) | 0.229 | 1.1 (1.02-1.18) | 0.013 | 1.08 (0.8-1.45) | 0.628 | 1.44 (0.65-3.2) | 0.372 | 1.04 (0.92-1.18) | 0.51 | 0.87 (0.56-1.35) | 0.547 |
| DM | 1.16 (1.1-1.23) | <0.001 | 1.18 (1.1-1.25) | <0.001 | 1.32 (0.91-1.91) | 0.142 | 2.45 (0.93-6.48) | 0.07 | 1.21 (1.03-1.42) | 0.021 | 1.3 (0.78-2.17) | 0.322 |
| Obesity | 0.86 (0.78-0.94) | 0.001 | 0.87 (0.78-0.96) | 0.007 | 0.87 (0.53-1.43) | 0.577 | 0.49 (0.07-3.59) | 0.48 | 0.89 (0.69-1.14) | 0.347 | 0.84 (0.36-1.92) | 0.677 |
| AF | 1.36 (1.29-1.43) | <0.001 | 1.37 (1.29-1.46) | <0.001 | 1.53 (1.14-2.07) | 0.005 | 1.36 (0.64-2.91) | 0.425 | 1.21 (1.05-1.38) | 0.007 | 1.03 (0.65-1.61) | 0.913 |
| MI | 0.89 (0.83-0.95) | 0.001 | 0.9 (0.84-0.97) | 0.005 | 0.21 (0.03-1.48) | 0.117 | 4.19 (0.57-30.94) | 0.16 | 1.6 (1.09-2.34) | 0.016 | 0.41 (0.06-2.94) | 0.374 |
| PCI | 0.73 (0.68-0.79) | <0.001 | 0.74 (0.69-0.81) | <0.001 | 1.04 (0.43-2.54) | 0.925 | 0 (0-Inf) | 0.998 | 0.87 (0.52-1.48) | 0.62 | 0.5 (0.07-3.6) | 0.492 |
| COPD | 1.42 (1.32-1.54) | <0.001 | 1.45 (1.33-1.59) | <0.001 | 1.4 (0.85-2.31) | 0.19 | 0 (0-Inf) | 0.997 | 1.32 (1.07-1.63) | 0.01 | 1.63 (1.06-2.53) | 0.027 |
| CKD | 1.7 (1.6-1.82) | <0.001 | 1.71 (1.59-1.84) | <0.001 | 1.63 (1.07-2.5) | 0.023 | 0.64 (0.09-4.72) | 0.662 | 1.83 (1.55-2.16) | <0.001 | 1.57 (0.91-2.71) | 0.107 |
| Anemia | 1.49 (1.41-1.58) | <0.001 | 1.55 (1.45-1.65) | <0.001 | 1.42 (1-2.03) | 0.051 | 1.28 (0.48-3.38) | 0.619 | 1.25 (1.1-1.43) | 0.001 | 1.58 (1-2.49) | 0.052 |
| SBP <120mmHg | 1.18 (1.1-1.27) | <0.001 | 1.13 (1.04-1.23) | 0.005 | 0.98 (0.68-1.41) | 0.907 | 0.45 (0.2-1) | 0.05 | 1.31 (1.12-1.53) | 0.001 | 1.59 (0.95-2.65) | 0.076 |
| SBP >130mmHg | 1.07 (1-1.15) | 0.039 | 1.12 (1.03-1.2) | 0.004 | 0.95 (0.64-1.42) | 0.814 | 0.39 (0.11-1.41) | 0.153 | 0.94 (0.79-1.12) | 0.473 | 0.75 (0.39-1.46) | 0.401 |
| Sodium <135mmol/L | 1.34 (1.24-1.46) | <0.001 | 1.42 (1.29-1.57) | <0.001 | 1.63 (1.02-2.61) | 0.04 | 1.25 (0.43-3.63) | 0.681 | 1.02 (0.84-1.23) | 0.869 | 1.4 (0.74-2.66) | 0.303 |
| Sodium >145mmol/L | 1.1 (0.98-1.25) | 0.117 | 1.22 (1.07-1.39) | 0.004 | 0.66 (0.27-1.62) | 0.369 | 1.17 (0.16-8.67) | 0.879 | 0.61 (0.41-0.9) | 0.013 | 1.88 (0.9-3.92) | 0.094 |
| Potassium <3.5mmol/L | 1.05 (0.96-1.16) | 0.291 | 1.02 (0.92-1.14) | 0.724 | 1.2 (0.71-2.05) | 0.494 | 0.97 (0.23-4.08) | 0.964 | 1.17 (0.94-1.46) | 0.152 | 1.38 (0.67-2.87) | 0.386 |
| Potassium >5.5mmol/L | 1.47 (1.18-1.83) | 0.001 | 1.51 (1.18-1.92) | 0.001 | 0.65 (0.09-4.62) | 0.664 | 0 (0-Inf) | 0.997 | 1.5 (0.85-2.65) | 0.163 | 1.42 (0.35-5.8) | 0.623 |
| HHF in previous 12months, heart failure hospitalization in previous 12 months; NYHA, New York Heart Association; LVEF, left ventricular ejection fraction; NT-proBNP, N-terminal pro-B-type; natriuretic peptide; RHR: rest heart rate; DM, diabete mellitus; MI, myocardial infarction; AF, atrial fibrillation; COPD, chronic obstructive pulmonary disease; CKD, chronic kidney disease; SBP, systolic blood pressure. | | | | | | | | | | | | |

**Table S8. Operational definitions and prioritization principles for HFpEF phenotypes**

| **Phenotype** | **Core clinical criteria** | **Prioritization rule** |
| --- | --- | --- |
| HFpEF-1 | CHD, hypertension, ischemia. etc related HF | Assigned if vascular disease was judged as dominant trigger |
| HFpEF-2 | HCM, amyloidosis, infiltrative CM. etc related HF | Takes precedence over vascular/arrhythmic causes |
| HFpEF-3 | PAH, RV dysfunction related HF | Assigned only if right-heart pathology predominated |
| HFpEF-4 | AF, valvular disease. etc related HF | Assigned if rhythm/valve disorder was primary cause |
| HFpEF-5 | Extracardiac disease related HF | Assigned if HF secondary to systemic disease |

CHD, coronary heart disease; HF, heart failure; HCM, hypertrophic cardiomyopathy; CM, cardiomyopathy; PAH, pulmonary arterial hypertension; RV, right ventricle; AF, atrial fibrillation.
